# Scaling-up strategies for controllable biosynthetic ZnO NPs using cell free-extract of endophytic *Streptomyces* *albus*: characterization, statistical optimization, and biomedical activities evaluation

**DOI:** 10.1038/s41598-023-29757-9

**Published:** 2023-02-23

**Authors:** Shahira H. EL-Moslamy, Mohamed S. Elnouby, Ahmed H. Rezk, Esmail M. El-Fakharany

**Affiliations:** 1 Bioprocess Development Department, Genetic Engineering and Biotechnology Research Institute (GEBRI), City of Scientific Research and Technological Applications (SRTA-City), New Borg Al-Arab City, 21934 Alexandria Egypt; 2 Advanced Technology and New Materials Research Institute (ATNMRI), City of Scientific Research and Technological Applications (SRTA-City), New Borg El‑Arab City, 21934 Alexandria Egypt; 3 Protein Research Department, Genetic Engineering and Biotechnology Research Institute (GEBRI), City of Scientific Research and Technological Applications (SRTA-City), New Borg Al-Arab City, 21934 Alexandria Egypt

**Keywords:** Biochemistry, Biotechnology, Microbiology

## Abstract

In this study, we identified a suitable precursor and good cellular compartmentalization for enhancing bioactive metabolites to produce biosynthetic zinc oxide nanoparticles (ZnO NPs). An effective medium for cultivating endophytic *Streptomyces* *albus* strain E56 was selected using several optimized approaches in order to maximize the yield of biosynthetic ZnO NPs. The highest biosynthetic ZnO NPs yield (4.63 g/L) was obtained when pipetting the mixed cell-free fractions with 100 mM of zinc sulfate as a precursor. The generation of biosynthetic ZnO NPs was quickly verified using a colored solution (white color) and UV–Visible spectroscopy (maximum peak, at 320 nm). On a small scale, the Taguchi method was applied to improve the culture medium for culturing the strain E56. As a result, its cell-dry weight was 3.85 times that of the control condition. And then the biosynthesis of ZnO NPs (7.59 g/L) was increased by 1.6 times. Furthermore, by using the Plackett–Burman design to improve the utilized biogenesis pathway, the biosynthesis of ZnO NPs (18.76 g/L) was increased by 4.3 times. To find the best growth production line, we used batch and fed batch fermentation modes to gradually scale up biomass output. All kinetics of studied cell growth were evaluated during fed-batch fermentation as follows: biomass yield was 271.45 g/L, yield coefficient was 94.25 g/g, and ZnO NPs yield was 345.32 g/L. In vitro, the effects of various dosages of the controllable biosynthetic ZnO NPs as antimicrobial and anticancer agents were also investigated. The treatments with controllable biosynthetic ZnO NPs had a significant impact on all the examined multidrug-resistant human pathogens as well as cancer cells.

## Introduction

Nanotechnology offers appealing technological tools for creating a wide range of innovative materials, and it appears to have enormous potential in the medical and biological sciences. Nanoparticles (NPs) are thought to be the starting point for the macro-structures of new materials and technologies^1,2^. One of the more intriguing methods appears to be the use of microorganisms in nanoparticle manufacturing. Microbially produced NPs have outstanding physical, chemical, optical, and electrical properties^3^. Nanoparticle fabrication is an important aspect of nanotechnology. A range of techniques, including physical and chemical approaches, can be used to synthesize NPs. These methods, however, are costly and require the use of toxic substances, as well as accumulation and limited nanoparticle stability. As a result, there is a critical need to explore nanoparticle fabrication techniques that are safe, eco-friendly, clean, and economical. Microbial-mediated NP synthesis is an innovative method with wide applications in agriculture, agricultural production, and medicine. Because of their toxicity, these NPs synthesized using traditional methods have limited therapeutic use. Because of the physio-chemical features of microbial-based NPs, this method also has the added benefit of prolonging the life of the NPs, which overcomes the limits of classic physical and chemical NPs synthesis methods.

ZnO is inorganic crystalline metal oxide^4^. The unique properties ZnO NPs make them suitable for a variety of applications, including; biological^5^, opto-electronics^6^, medicinal^7^, energy storage^8^, and electrochemical applications. Until now, ZnO NPs could be synthesized via several methods, including; thermal evaporation technique^9^, electrodeposition^10^, and hydrothermal reaction^11^. Depending on preparation conditions, the resulting nanostructured ZnO NPs had different morphological and crystalline structures. Thus, controlling the morphological and crystallographic properties of any nanomaterial is a key factor in enhancing its performance in such applications. ZnO NPs is a polar crystal that presents anisotropic growth behavior due to various crystal facets with different growth directions^12^. Ongoing efforts have been made to improve the structure of the manufactured NPs in order to enhance their performance in wide variety of applications. The two main variables that affect how the structure of NPs is controlled are the capping agent and surface energy. The structure of ZnO NPs has been controlled hydrothermally, according to Najib et al^13^. Accordingly, the morphology of ZnO NPs electrodes using high-performance supercapacitor devices can be adjusted to control the problem of ZnO NPs structures. There are several applications for ZnO NPs, including in the production of ceramics, solar cells, electronics, photocatalysis, and electrotechnology^14–16^. Because of their outstanding UV-absorption potential, ZnO NPs are utilized in cosmetics and sunscreen products. In addition to their antimicrobial and deodorizing properties, ZnO NPs are used to increase the fabrics' UV tolerance^17^. Recent research has demonstrated that ZnO NPs may be used as food additives to boost growth performance, reduce oxidative stress, and reduce inflammation^18^. Additionally, they can enhance egg quality and layer chicken production^19^. Because the Food and Drug Administration has certified ZnO NPs as a safe product, they can be used by both animals and humans^20,21^. Zinc, a vital trace element found in all biological tissues and an essential part of the majority of enzyme systems, is also present in all biological tissues. Therefore, it plays a role in the body's metabolism and is assimilated during nucleic acid and protein synthesis, neuronal cell development, and hematopoiesis^21^. Various organisms can be utilized to safely biosynthesize nanomaterials, including yeast, plants, fungus, and bacteria^22,23^. Inorganic NPs can be biosynthesized intracellularly or extracellularly by both unicellular and multicellular microorganisms^24^. Synthesis of green NPs employing microorganisms has been extensively studied for numerous nanomaterials^20,25^. It is unclear exactly how macro, and microcells convert metallic ions into metal oxide NPs. For the synthesis of ZnO NPs, microbial methods are preferable to tissue cultures or employing plant sources due to their scalability, efficiency, and microorganism life cycle^26^. Because of their high biomass production, quick growth, and environmental friendliness, actinomycetes, particularly *Streptomyces*, are one of the main candidates for the biological synthesis of NPs on a large scale and at low cost. This *Streptomyces* sp., is a Gram-positive filamentous bacterial genus known for producing a huge number of secondary metabolites. *Streptomyces* are of interest for the industrial production of most microbial metabolites due to their non-pathogenic nature for humans^27^. According to the literature, *Streptomyces* sp. is frequently recognized as the main source of medicines used in human and veterinary applications.

Endophytes are a group of microorganisms, including bacteria, fungi, and actinomycetes that can colonise inside plant tissues without causing any harmful symptoms of disease in the host plant^28^. Numerous endophytic microbes' metabolites, particularly those produced by actinomycetes, can be employed as reducing, capping, and stabilizing agents in the synthesis of NPs^29^. In comparison to endophytic actinomycetes, the synthesis of various NPs by endophytic fungi and bacteria has received substantial investigation. These bacterial and fungal' activities and metabolites have previously been studied to assess their potential for biological and biotechnological applications^30^. One of the few endophytic actinomycete species that are used as biomaterials in the manufacture of Ag-NPs are the endophytic *Streptomyces* *antimycoticus* L-1^31^. It has been claimed that marine endophytic actinomycetes like *Streptomyces* *capillispiralis* can effectively generate Cu NPs smaller than 100 nm^32^. Generally, this endophytic strain could generate internal and extracellular active metabolites, including proteins, amino acids, and enzymes, which are essential for the production of metal oxide NPs. These active molecules act as ion reducing, metal capping, and stabilizing agents during the manufacturing of metal NPs^20,33^. Endophytic *Streptomyces* *albus* has received minimal attention for use in pharmaceutical applications employing a production line of nanobiotechnology. Therefore, in our investigation, we decided to use it as a safe bio-factory for bio-synthesizing ZnO NPs. To the best of our knowledge, this is the first study to describe the biosynthesis production of ZnO NPs using a local endophytic strain of *Streptomyces* *albus.* This is accomplished by using a variety of bioprocessing techniques to increase our *Streptomyces* biomass and its ZnO NPs yield through a rapid biosynthesis reaction. The key biosynthesis parameters, such as time, pH, and extract ratio, were statistically optimized to control the size, shape, and dry weight of biosynthesized NPs^34–36^. Experimental designs have become popular in recent years for the optimization of statistical approaches since they require less time, effort, and resources than traditional techniques^37–40^. It's a fantastic tool for evaluating several variables at multiple levels in a small number of experiments. Two types of experimental designs are utilized for technique optimization: screening and optimization design (response surface methodology). The screening design employs a full factorial design, such as Taguchi Robust Design (TRD) and Plackett–Burman Design (PBD) to determine the most significant independent variable influencing response^41^. A scale-up method is required to maximize the bulk yields of bioactive metabolites generated in industrial applications^42^. As a result, it is preferable to evaluate appropriate operating parameters in a bench-scale bioreactor before generating them in a semi-industrial large-scale bioreactor. In a stirred-tank bioreactor, the aeration rate and agitation speed are expected to be significant factors in biomass production and, as a result, metabolite production^43^. Aeration and agitation help to stabilize the uniformity of chemical and physical conditions in the fermentation media, in addition to permitting improved mass and oxygen transportation between phases. This is critical, especially for insoluble substrates, because it affects microbial culture availability and substrate consumption, and thus metabolite synthesis^44^.

The aim of this study is to investigate the feasibility of synthesizing ZnO NPs using ZnSO_4_^.^7H_2_O as a precursor and an endophytic *Streptomyces* *albus* cell-free extract as a reducing/capping agent. Another important goal is to characterize the biosynthesized NPs using UV–Vis, FTIR, XRD, TEM, SEM, EDX, and XPS approaches. The biosynthesized ZnO NPs were scaled up using fed-batch fermentation methods and statistically generated experimental designs. Finally, the biological activities of the biosynthesised ZnO NPs were assessed for their potential biomedical applications, including their cytotoxic effects on normal and malignant cells, as well as their antibacterial efficacy against pathogenic Gram-positive and Gram-negative bacteria and unicellular fungi.

## Results and discussion

In order to biosynthesize metallic NPs with different morphologies (shape and size), a mixture of biomolecules such as enzymes, proteins, and peptides produced by microorganisms or even the phytochemicals that are extracted from plants is extensively applied^45–47^. In recent years, this method has also proven to be useful for the production of nanomaterials with various morphologies. It has recently been demonstrated that processing parameters in the biosynthetic reaction, such as temperature, pH, mixing ratio, incubation period, and aeration, can enhance the biosynthesis of NPs with different morphologies^20,48^. Our study focuses on the optimization of the ZnO NPs biosynthetic reaction and the microbial growth culturing process using a safe microbial source, such as endophytic *Streptomyces* *albus* strain E56. The fed-batch fermentation method, which is explained in more detail below, was used to scale up the biosynthesized ZnO NPs.

### Surveys to detect the best effective cell-free extract as a reductant and acceptable precursor for the biosynthesis of ZnO NPs as antimicrobial agents

**Figure 1:**
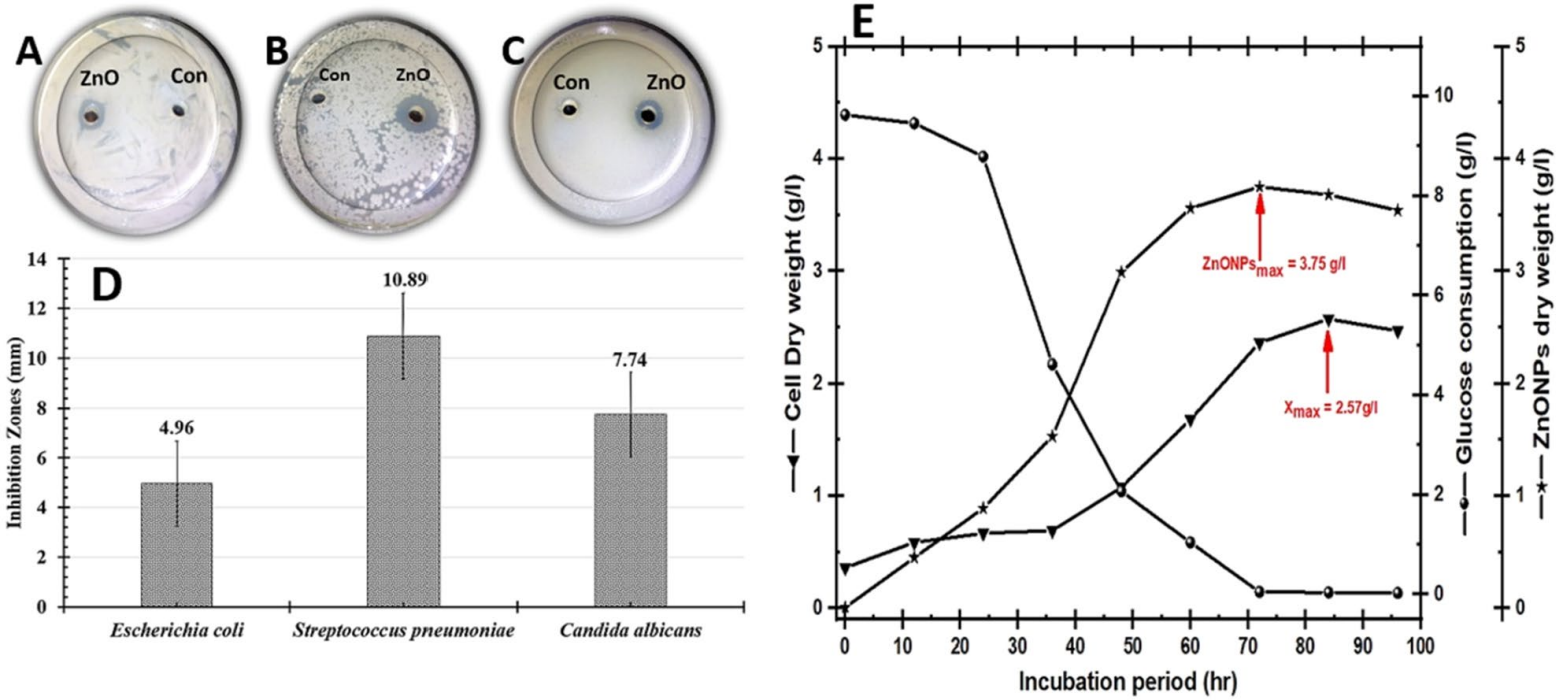
Represents the behavior of the biosynthetic ZnO NPs using mixed cell-free extract of strain E56 as a reductant/capping, and Zn (CH_3_ COO)_2_.2H_2_O as a precursor. Antimicrobial survey for biosynthetic ZnO NPs against **(A)** *Escherichia* *coli,* **(B)** *Staphylococcus* *aureus,* and **(C)** *Candida* *albicans.* The extract of strain E56 was used as a control (Con). Additionally, histogram shows the calculated inhibition zones (**D**). Real-time monitoring of strain E56's growth pattern (g/L) and the ZnO NPs it generates (**E**).

This section looked into the possibilities of employing Zn (CH_3_COO)_2_^.^2H_2_O as a precursor for the biosynthesis of ZnO NPs using the biomass filtrates of *Streptomyces* *albus* strain E56 as a reducing/capping agent. Following the experimental period, the extracted bioactive metabolites with different active groups converted Zn^2+^ (colorless) into ZnO NPs (white turbidity). Then, the antimicrobial properties of biosynthetic ZnO NPs against three multidrug-resistant pathogens, including *Escherichia* *coli*, *Staphylococcus* *aureus*, and *Candida* *albicans* were studied utilizing an agar-well diffusion experiment. As shown in Fig. 1D the biosynthetic ZnO NPs inhibited the growth of all tested pathogens. The biggest inhibitory zone was found in *Staphylococcus* *aureus*, reaching 10.89 mm (Fig. 1B), followed by *Candida* *albicans*, measuring 7.74 mm (Fig. 1C). The smallest inhibitory zone was found in *Escherichia* *coli*, at 4.96 mm (Fig. 1A). Our findings are in line with prior studies since the biosynthetic ZnO NPs have antibacterial ability against both gram-positive and gram-negative bacteria as well as unicellular fungi^33,49^.

**Figure 2:**
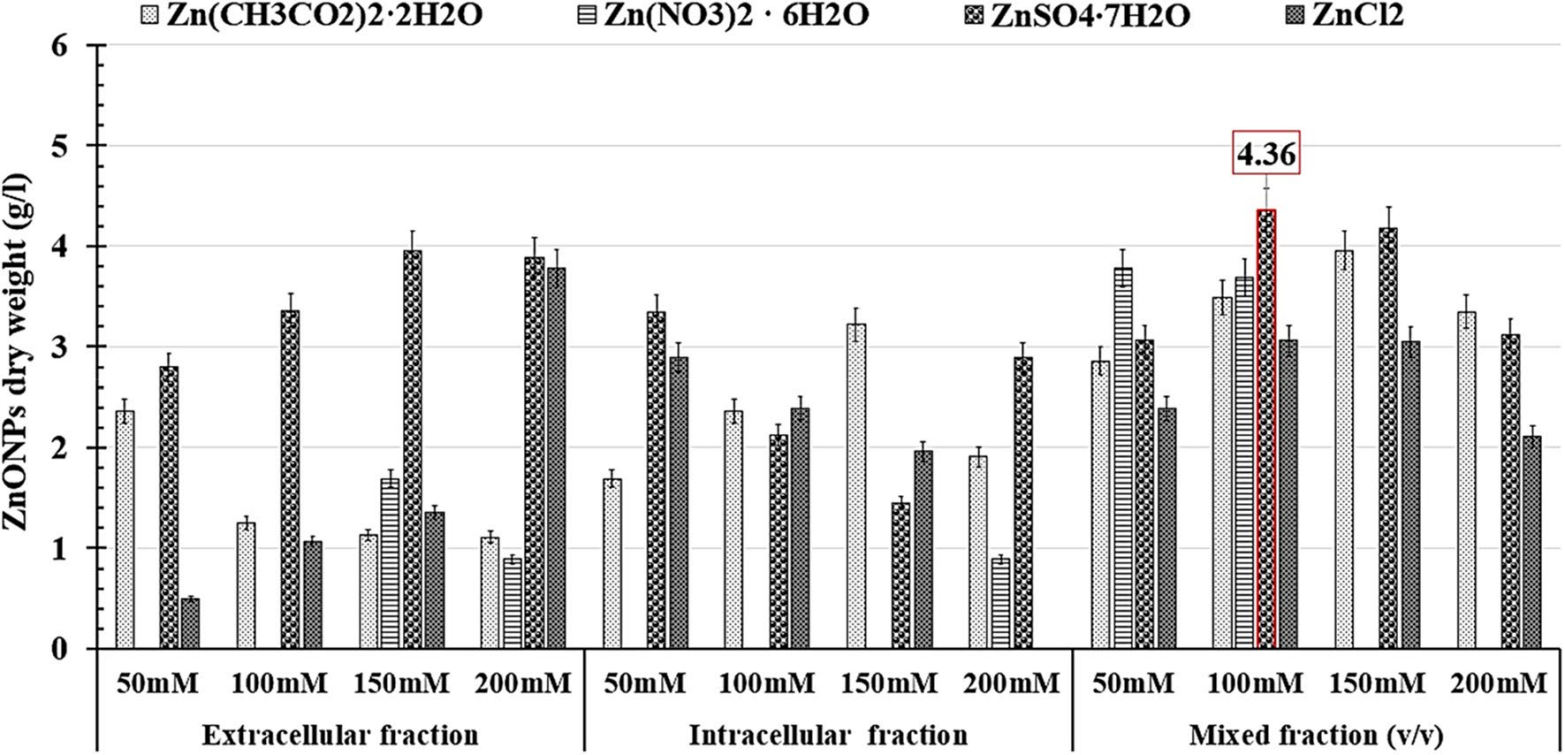
Represents the maximum yield of biosynthetic ZnO NPs using Zn (CH_3_ COO)_2_^.^2H_2_O, Zn (NO_3_)_2_^.^6H_2_O, ZnSO_4_^.^7H_2_O, and ZnCl_2_ as a precursor at 50, 100, 150, and 200 mM, that mixed with the extracellular and intracellular fractions separately or mixed (v/v) as a reductant/capping agent.

According to the majority of research studies, the nanoparticle biosynthesis process on a lab scale is well documented and explained. Unfortunately, there are no publications that clarify the techniques for scaling up the nanoparticle biosynthesis process to a semi-industrial scale using large-scale strategies. This is due to the difficulty in producing NPs of the same quality and quantity, particularly when biological sources are used^41^. A scaling-up production strategy is an important step in converting achievements in nanobiotechnology processes from scientific publications into real technologies and commercial products. As a result, in order to solve a large-scale challenge, the scaling-up strategy for biosynthesis of NPs, which includes increasing the quality and quantities of nanoproducts based on laboratory scale results, requires more optimization, development, and validation^50^. As a response, we proposed a distinct plan to improve, develop, and validate ZnO NPs biosynthesis from the lab to the semi-industrial scale in our work. As shown in Fig. 1E, we first monitored the growth of the endophytic *Streptomyces* *albus* strain E56 to determine the maximum cell dry weight (X_max_) that produced the greatest quantity of biosynthetic ZnO NPs. The X_max_ was found to be 2.57 g/L after 84 h of incubation period. In order to determine the best time for ZnO NPs biosynthesis, the biomass that was harvested during that incubation period was used separately. After 72 h, the maximum dry weight of ZnO NPs (P_max_) was 3.75 g/L (Fig. 1E). The internal and extracellular cell-free fractions were subsequently used singly or in combination (v/v) as a reductant/capping agent to optimize the biosynthetic ZnO NPs. Different precursors, including Zn (CH_3_ COO)_2_^.^2H_2_O, Zn (NO_3_)_2_^.^6H_2_O, ZnSO_4_^.^7H_2_O, and ZnCl_2_, were examined all through the same tests at concentrations of 50, 100, 150, and 200 mM, as shown in Fig. 2. The maximum dry weight of biosynthetic ZnO NPs (4.63 g/L) were obtained from the mixed cell-free fraction when it was pipetted with 100 mM of ZnSO_4_^.^7H_2_O, as seen in Fig. 2.

**Figure 3:**
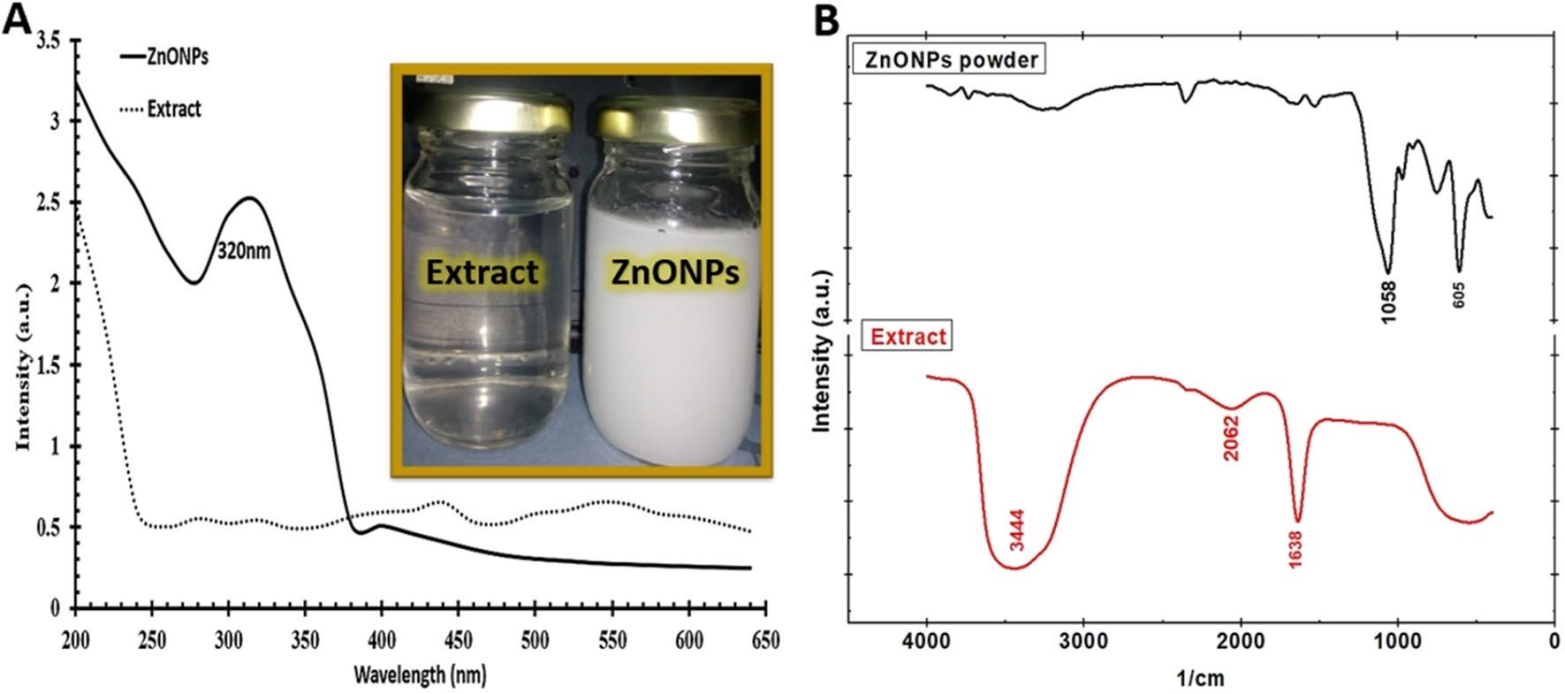
(**A**) UV–visible absorption spectra of ZnO NPs and cell-free mixed extract; **(B)** FT-IR spectroscopy of used mixed extract and biosynthesized ZnO NPs; besides the photos appeared the changing color of mixed cell-free extract into white color that confirmed the bio-synthesis process of ZnO NPs.

The formation of biosynthetic ZnO NPs was rapidly confirmed using a colored solution as well as UV–visible spectroscopy (Fig. 3A). The differences in colors show that Zn ions have been reduced to yield biosynthetic ZnO NPs. Therefore, in our research, the optical productivity of these biosynthetic ZnO NPs was assessed by observing the change in color of a cell-free extract from colorless to white. The mixture's color change was caused by the surface plasma resonance (SPR) phenomenon, which was carried on by the bio-metabolites' generation of ZnO NPs, according to previous reports. This white color is common for ZnO NPs, which are equivalent to earlier biosynthetic ZnO NPs generated from other bioactive materials and have a visible range of 300–400 nm^51^. The real highest absorption peak of synthetic ZnO NPs that verified the generation of ZnO NPs previously ranged from 275 to 365 nm, depending on the biosynthesis technique and reduction reaction (reductant and precursor) utilized^20,33,52–55^. For our biosynthetic ZnO NPs, the maximum UV–visible absorption peak was found at 320 nm.

The functional groups available in the cell-free extract were identified by FT-IR and compared to the wavenumber of our biosynthetic ZnO NPs, as shown in Fig. 3B. The FT-IR spectroscopy technique can be used to identify and characterize the chemical structure of any functional groups that acted as reducing and capping agents during the biosynthesis of ZnO NPs. Several sharp peaks were observed in the cell-free extract spectrum, including the one reported at 1638, which refers to the graphitic domain of C=C (alkenes) and C=N (aromatic amines)^56^. Furthermore, the detected peak at 2062 is linked to C=O vibrations (carbonyl stretch of a carboxylic acid, ketones, aldehydes, or ester). While the observed peak at 3444 is associated with the stretching vibration of (O–H stretch), which refers to alcohols and phenols^57^. All of these peaks are eliminated in the biosynthetic ZnO NPs spectrum, as seen in Fig. 3B. The detected peak at 605, which corresponds to the stretching vibration of ZnO NPs, identified and validated the biosynthetic ZnO NPs^18,24,58^. Furthermore, the detected signal at 1058 is referred to C–O–C stretching mode ^59^ as an aliphatic ether or aldehyde, which may act as a stabilizing agent. Therefore, the availability of these functional chemical groups in cell-free extract, which regulate the reduction of Zn ions to ZnO NPs, could explain the generation of ZnO NPs. Based on the results of a recent study^20^, Eq. (1) provides a hypothesized biological synthesis pathway for ZnO NPs using mixed cell-free extract as a reductant/capping agent and NaOH to control the biosynthesis reaction pH that was mixed with ZnSO_4_.7H_2_O as a precursor.

$${\mathrm{ZnSO}}_{4}\cdot 7{\mathrm{H}}_{2}\mathrm{O}+2\mathrm{NaOH}+Cell-free\,extract \to {\mathrm{Zn}\left(\mathrm{OH}\right)}_{2 \left(White\,precipitate\right)}\downarrow +{\mathrm{Na}}_{2}{\mathrm{SO}}_{2}$$

$${\mathrm{Zn}(\mathrm{OH})}_{2}+{2\mathrm{H}}_{2}\mathrm{O }{\mathrm{Zn}(\mathrm{OH})}_{4}^{2-}\downarrow +2{\mathrm{H}}^{+}$$

1 $${\mathrm{Zn}\left(\mathrm{OH}\right)}_{4}^{2-}\mathrm{ZnO}+2\mathrm{OH}+{2\mathrm{H}}_{2}\mathrm{O}\to \mathrm{ZnO }NPs \downarrow$$

According to other publications, the production of ZnO NPs was thought to be initiated by the interaction of zinc molecules from salt precursors with oxygen from functional groups within cell-free extract^26^. Other earlier studies also suggested that the presence of amines, phenols, proteins, hydroxyl, and carboxylate groups contributed to the reduction process^31,39,60^. Others proposed that, the reductase enzyme was generated by microbes in growth conditions and could carry out the extracellular mechanism of ZnO NPs^41^. Subsequently, the Zn^2+^ was reduced to Zn^o^, which generates ZnO NPs utilizing nicotinamide adenine dinucleotide plus hydrogen ion-dependent reductase enzymes as electron carriers. Then, metal ions were reduced to nanoscales while the reductase enzymes acquired electrons from NADH, which were then oxidized to NAD+^33^.

### Statistical optimization for maximizing cell dry weight of endophytic *Streptomyces albus* strain E56 dry using Taguchi design

Biogenic or green NPs are promising antimicrobial and anticancer agents for more safe, targeted, and cost-effective pharmaceuticals or drug delivery vehicles^23^. The presence of various metabolites, such as proteins, enzymes, and other biomolecules that act as reducing, capping, and stabilizing agents, is attributed to the biogenic synthesis of ZnO NPs by biological systems^46,49^. The secreted metabolites are related to the different shapes, sizes, dispersion, and stability of ZnO NPs. The *Streptomyces* genus is distinguished for its complicated life cycle, which includes morphological and developmental stages that influence metabolite formation. The optimization of *Streptomyces* *albus* fermentation conditions for the manufacturing of specific bioactive molecules, as well as the scaling-up of the biotechnological process in a bioreactor with controlled parameters, are also areas that have yet to be explored^61^. In order to maximize productivity and growth yields, incubation temperature, pH, medium composition, oxygen levels, aeration, and agitation should all be optimized in the design of a biotechnological manufacturing process of industrially interesting chemicals^62,63^.

In this study, an integrated optimization of key fermentation processing parameters was employed for the first time to maximize the production of bioactive metabolites by the strain E56. On a small scale, preliminary NMM medium components were optimized using Taguchi experimental design to maximize the produced bioactive metabolites that would be used as reducing/capping agents in biosynthetic ZnO NPs.

**Figure 4:**
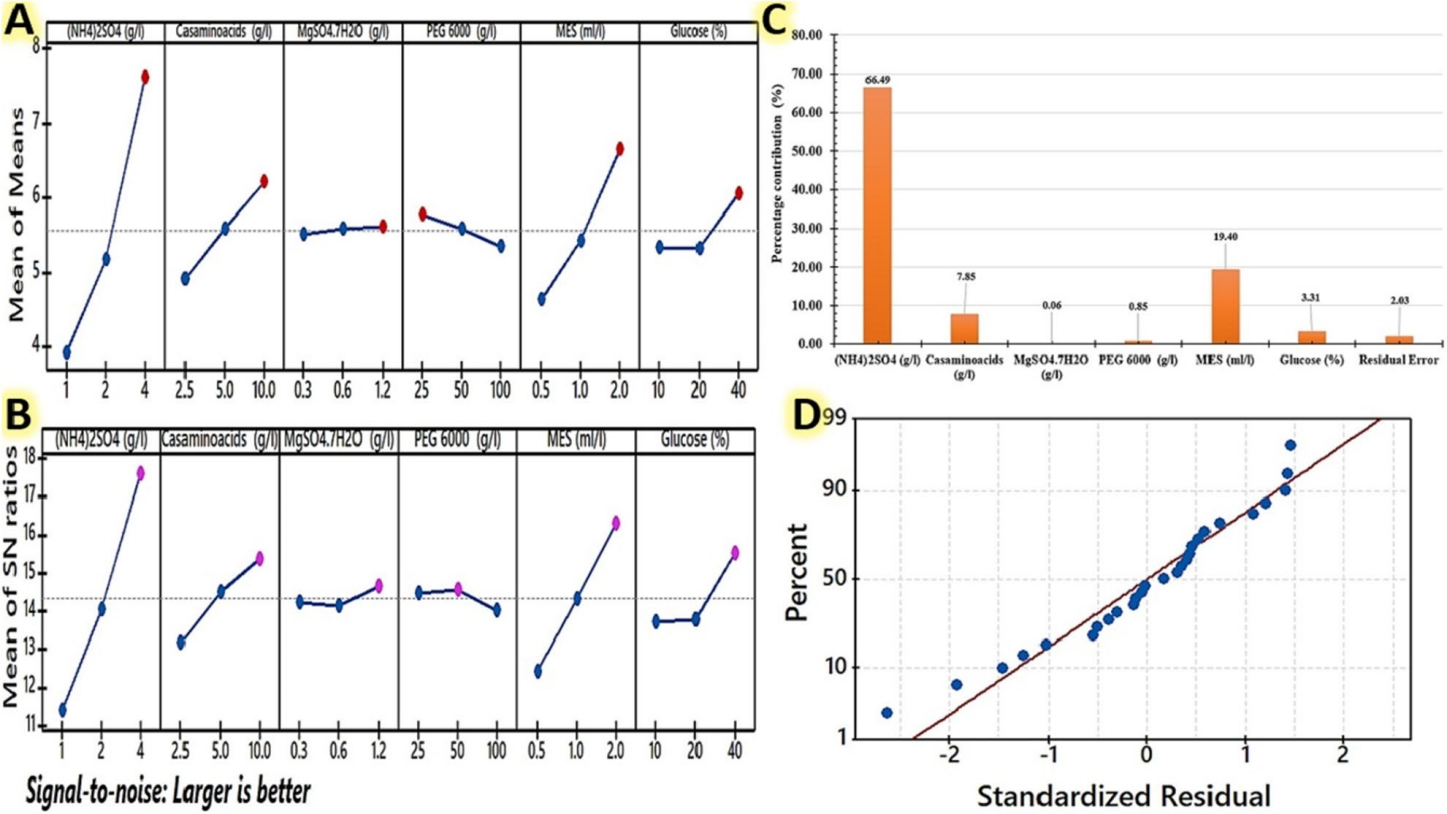
Characterizes Taguchi's experimental results; (**A**) the main effects plot for the mean of produced cell dry weight of strain E56; (**B**): the larger-the-better main effects plot for S/N ratios, with the horizontal axis indicating the different levels of each significant component and the lines representing the trend of each factor with respect to different levels. (**C**): the percentage contribution of each factor to the cell dry weight yield. (**D**): the residual normal probability plot.

Genichi Taguchi has designed a visually attractive and cost-effective tool for maximizing and developing high-quality industrial production processes. Taguchi's model includes a number of arrays that can be employed depending on the needs of the experiment. The design of an orthogonal array (OA) is coded as Ln (mk), where n is the total number of sections, m means the number of parameter levels, and k shows the number of parameters^40^. To maximize the dry cell mass weight (g/L) of the strain E56, we employed Taguchi's L_27_ (3^6^) orthogonal array design. As shown in Table 1, 27 trials were carried out with six factors classified based on the L_27_ (3^6^) OA design in order to determine the produced cell dry weight and S/N ratio values. As indicated in Table 2, the experimental data can be evaluated using an ANOVA as well as an F-test. The model we used had an R^2^ of 0.97, indicating that the experimental data was well-fitting. The model, as well as the parameters in the model, were extremely significant (P < 0.0001). The adjusted R^2^ value of 0.96 was fairly close to the predicted R^2^ value of 0.94 (Table 2). The F-value of the model is 113.52, while the significance F-value is 2.23E−14. There was only a 1.041% chance that the model F-value would be high due to noise. The presence of residuals above and below the residual plot's zero-line suggested that the proposed model was sufficient. The residual plots for SN ratios and standard deviations are shown in Fig. 4D. The residual plots reveal that the residuals follow a straight-line distribution, indicating that the model is well-fit to the data. Because the response plot is a straight line, the errors are assumed to be normally distributed. According to the *P*-values, (NH_4_)_2_SO_4_, casamino acids, and MES were significant factors, followed by glucose and PEG 6000. Equation (2) represents this orthogonal array model, which describes the correlations between the six factors and produced the dry cell mass weight.

**Table 1:** Experimental setup for maximization the dry cell mass weight of strain E56 using Taguchi's L_27_ (3^6^) orthogonal array design.

Runs	(NH4)_2_SO_4_ (g/L)	Casamino acids (g/L)	MgSO_4_.7H_2_O (g/L)	PEG 6000 (g/L)	MES (mL/l)	Glucose (%)	Dry cell mass weight (g/L)		S/N ratio (dB)
							Experimental values	Predicted values	
L1	1	2.5	0.3	25	0.5	10	2.35	2.28	7.42
L2	1	2.5	0.3	25	1	20	2.99	3.21	9.51
L3	1	2.5	0.3	25	2	40	4.89	5.05	13.79
L4	1	5	0.6	50	0.5	10	2.68	2.59	8.56
L5	1	5	0.6	50	1	20	3.56	3.52	11.03
L6	1	5	0.6	50	2	40	5.65	5.36	15.04
L7	1	10	1.2	100	0.5	10	2.69	3.21	8.60
L8	1	10	1.2	100	1	20	4.38	4.13	12.83
L9	1	10	1.2	100	2	40	6.09	5.98	15.69
L10	2	2.5	0.6	100	0.5	20	2.736	3.39	8.74
L11	2	2.5	0.6	100	1	40	4.76	4.58	13.55
L12	2	2.5	0.6	100	2	10	5.44	5.13	14.71
L13	2	5	1.2	25	0.5	20	4.45	4.29	12.97
L14	2	5	1.2	25	1	40	5.5	5.47	14.81
L15	2	5	1.2	25	2	10	6.36	6.02	16.07
L16	2	10	0.3	50	0.5	20	4.95	4.88	13.89
L17	2	10	0.3	50	1	40	5.76	6.06	15.21
L18	2	10	0.3	50	2	10	6.56	6.61	16.34
L19	4	2.5	1.2	50	0.5	40	6.96	6.72	16.85
L20	4	2.5	1.2	50	1	10	6.96	6.60	16.85
L21	4	2.5	1.2	50	2	20	7.15	8.19	17.09
L22	4	5	0.3	100	0.5	40	6.75	6.75	16.59
L23	4	5	0.3	100	1	10	6.96	6.64	16.85
L24	4	5	0.3	100	2	20	8.34	8.23	18.42
L25	4	10	0.6	25	0.5	40	8.12	8.03	18.19
L26	4	10	0.6	25	1	10	7.96	7.92	18.02
L27	4	10	0.6	25	2	20	9.36	9.50	19.43

**Table 2:** Analysis of variance (ANOVA) results for Taguchi design experimental design results used to maximize the cell dry weight of strain E56.

	Coefficients	Standard error	t stat	P-value	
Intercept	−0.179481481	0.333656046	−0.53792366	0.596565641	
(NH4)_2_SO_4_ (g/L)	1.231460317	0.057035453	21.59113764	2.49248E−15***	
Casamino acids (g/L)	0.165574603	0.022814181	7.257529933	5.07357E−07***	
MgSO_4_^.^7H_2_O (g/L)	0.112010582	0.190118177	0.589162929	0.562348473	
PEG 6000 (g/L)	−0.005530159	0.002281418	−2.424000526	0.024953359*	
MES (mL/l)	1.327396825	0.114070906	11.63659403	2.33637E−10***	
Glucose (%)	0.025907937	0.005703545	4.542426703	0.00019819**	

ANOVA					
	*df*	*SS*	*MS*	*F*	Significance *F*
Regression	6	93.05935714	15.50989286	113.5193029	2.23422E−14
Residual	20	2.732556042	0.136627802		
Total	26	95.79191319			

Multiple R = 0.96, R^2^ = 0.97, AdjR^2^ = 0.96, R-sq(pred); 0.94, SE = 0.37.

*Significant values.

2 $$Dry\,cell\, mass\, weight \left(g/L\right)=-0.17+1.232 {\left({\mathrm{NH}}_{4}\right)}_{2}{\mathrm{SO}}_{4}+0.166 Casamino\,acids+0.122{\mathrm{MgSO}}_{4}{.7\mathrm{H}}_{2}\mathrm{O}-0.005\,PEG\,6000+1.327\mathrm{ MES}+0.0259\,Glucose$$

Based on the ANOVA analysis of the S/N ratio value and the calculation of the main effect for cell dry weight using factor level, the top rank really does have the highest S/N ratio value (larger is better), as shown in Fig. 4A. A primary impact graphic was constructed for the data means obtained during the optimization trial runs (Fig. 4B). The correlation between the factors and their responses in the form of generated cell weights was shown using the main effect plots. With the exception of PGE 6000 and MgSO_4_^.^7H_2_O, raising all examined variables from level 1 to level 3 resulted in an increase in cell dry weight. According to the results, adjusting these variables has a significant impact on the dry weight (g/L) of the cells. On the other hand, PGE 6000 and MgSO_4_^.^7H_2_O are less important parameters for determining the dry cell weight achieved. The dry cell weight decreases when the concentration of PGE 6000, MgSO_4_^.^7H_2_O rises. The parametric percent contribution of various factors in the total production of dry cell weight is shown in Fig. 4C. As can be observed, the parameter (NH_4_)_2_SO_4_ has the highest percentage contribution of 66.49%, followed by MES (19.40%), then casamino acids (7.85%). While glucose contributes the smallest percentage of the whole effect, at 3.31%.

The ranked variables with actual values from the most to the least impactful parameters that affect the produced cell dry weight of strain E56 were achieved with 4 g of (NH_4_)_2_SO_4_, 10 g of casamino acids, 1.2 g of MgSO_4_^.^7H_2_O, 50 g/L of PEG (6000), 2 mL/l of MES (solution B), and 40% glucose. Finally, to validate the appropriate medium ingredient concentrations for improving cell dry weight production, a confirmation test for this design is necessary. The best concentrations for each factor were calculated, and a specific number of trials were conducted under these conditions. Based on the variables and their levels measured, the result of the confirmation experiment (mean: 9.56 g/L and S/N ratio: 22.19 dB) is compared to the expected value (mean: 9.88 g/L and S/N ratio: 22.256 dB), suggesting that the predicted and experimental values are relatively close. The strain E56's cell dry weight can be increased and improved using the Taguchi approach (3.85 times greater than the basal condition). The synthesis of ZnO NPs (7.59 g/L) increased by 1.6 times when a cell-free extract of the maximum dry cell mass was employed in the biosynthetic ZnO NPs pathway as compared to the basal condition (4.36 g/L).

### Maximizing the yield of the biosynthetic ZnO NPs using Plackett–Burman experimental design

**Figure 5:**
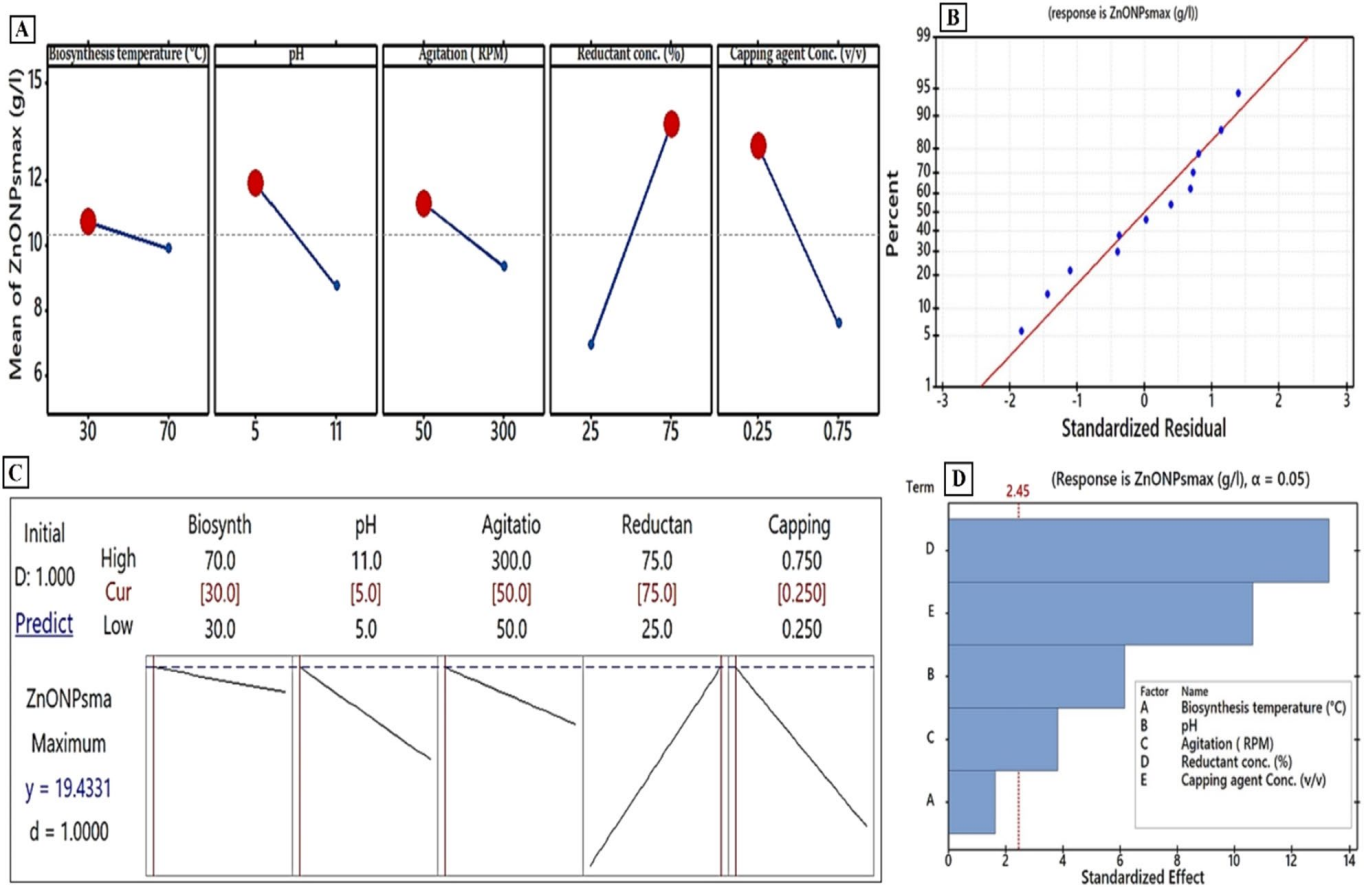
(**A**) The main effect plot of each evaluated variable on the dry weight of the biosynthetic ZnO NPs using Plackett–Burman design (**C**) a response optimizer with an expected control system for the ZnO NPs bio-synthesis reaction, with a maximum outcome and optimal values of variables investigated. (**B**) A residual normal probability plot. (**D**) The Pareto chart of standardized effects.

In the previous sections, we picked a suitable precursor, attractive cell compartmentalization for bioactive metabolites, and an ideal medium for growing the strain E56 to increase the dry weight of the biosynthetic ZnO NPs. Many other factors could influence the biosynthetic ZnO NPs reaction. In order to increase the yield of ZnO NPs, major biosynthetic reaction parameters such as temperature of biosynthesis (°C), pH, agitation (RPM), reductant concentration (%), and capping agent concentration (v/v) were also evaluated in our study. The effect of the aforementioned five parameters on the biosynthetic ZnO NPs yield was statistically analyzed using PBD. This design was used to develop the biosynthesis reaction components and biosynthesis settings that affect the biosynthesis reaction of ZnO NPs. The response was calculated using the dry weight of ZnO NPs (g/L). The results of the 12-run experimental setup using actual values for the parameters under investigation are displayed in Table 3. As is noticeable, the yield of biosynthetic ZnO NPs ranged from 2.56 to 17.36 g/L. Notably, the highest yield of ZnO NPs dry weight (17.36 g/L) was produced in run Z10, and the lowest yield (2.56 g/L) was achieved in run Z12. This design's model F-value and significance F-value were reported as 68.937 and 3.22E−05, respectively, indicating that they are significant and "p-value > F" values less than 0.050. Table 4 shows the results of a statistical analysis of the experimental data using the F-test for ANOVA. The model's significance is shown by the model F value for ZnO NPs dry weight, which implies that there was a correlation between the response and the independent factors. Additionally, it reveals that the independent variables in the suggested model improved model fit. The probability value was used to establish the significance of each independent variable; thus, a p-value < 0.05 shows that factor is significant. R^2^ has always been in the 0 to 1 range. If the R^2^ is close to 1, the model is stronger and the predicted response is excellent^40^. Our design's coefficient of determination (R^2^ = 0.9829) demonstrated that the independent factors can explain 98.29% of the variation in the ZnO NPs biosynthesis process, while only 1.71% is unaccounted for. A model with a very high adjusted coefficient of determination (Adj. R^2^ = 96.86%) is similarly highly significant. The "Pred R^2^" value of 93.16% is close to the "adj R^2^" value of 96.86%. This shows a significant correlation between predicted and experimental ZnO NP yields. Additionally, the normal probability plot was illustrated, as shown in Fig. 5B. The residuals normal probability plot can be used to discover and demonstrate systematic deviations from normality^64^. The spots along the diagonal line in this plot of internally observed values indicate that the data is normally distributed and that the model fits the experimental results well. Based on estimated coefficients and multiple regression analysis of the experimental data, the first-order model equation (Eq. 3) was used to connect the test variables and the response variable.

**Table 3:** Twelve trials of Plackett–Burman design to detect significant independent factors that influence ZnO NPs dry weight yield from the strain E56 cell-free extract.

Run	Biosynthesis temperature (°C)	pH	Agitation (RPM)	Reductant conc. (%)	Capping agent Conc. (v/v)	Dry weight of ZnO NPs (g/L)		Residuals	Standard residuals
						Actual values	Predicted values		
Z1	70	5	50	25	0.75	6.1	6.33	−0.24	−0.36
Z2	70	11	50	75	0.75	10	9.98	0.01	0.02
Z3	30	11	50	25	0.25	9.89	9.46	0.43	0.66
Z4	30	5	300	75	0.75	12.9	12.02	0.87	1.33
Z5	70	11	300	25	0.75	0.97	1.22	−0.25	−0.38
Z6	30	11	300	75	0.25	13.17	14.32	−1.15	−1.75
Z7	30	5	50	25	0.25	12.87	12.62	0.25	0.38
Z8	30	5	50	75	0.75	13.07	13.98	−0.91	−1.38
Z9	70	5	300	25	0.25	9.14	9.83	−0.69	−1.06
Z10	70	5	300	75	0.25	17.36	16.64	0.72	1.09
Z11	70	11	50	75	0.25	15.89	15.44	0.45	0.69
Z12	30	11	300	25	0.75	2.56	2.05	0.51	0.77

ANOVA						Regression statistics			
	*df*	*SS*	*MS*	*F*	Significance *F*	Standard error	*R-sq*	*R-sq(adj)*	*R-sq(pred)*
Regression	5	271.88	54.376	68.937	3.22E−05	0.89	98.29%	96.86%	93.16%
Residual	6	4.733	0.789						
Total	11	276.6

**Table 4:** Analysis of variance of Plackett–Burman design for biosynthesis reaction for ZnO NPs using cell-free extract of strain E56.

Source	DF	Adj SS	Coefficients	Standard error	*t* Stat	*P*-value	Adj MS	F-value
Model	5	271.882					54.376	68.94
Linear	5	271.882					54.376	68.94
Intercept			15.5981	1.2644	12.3365	1.73E−05		
Biosynthesis temperature (°C)	1	2.093	−0.0209	0.0128	−1.6288	0.154479097	2.093	2.65
pH	1	29.968	−0.5268	0.0855	−6.1638	0.00083703**	29.968	37.99
Agitation (RPM)	1	11.456	−0.0078	0.0021	−3.811	0.008853564**	11.456	14.52
Reductant conc. (%)	1	139.091	0.1362	0.0103	13.2791	1.13E−05***	139.091	176.34
Capping agent conc. (v/v)	1	89.275	−10.9103	1.0255	−10.6386	4.07E−05***	89.275	113.18
Error	6	4.733					0.789	
Total	11	276.615						

*Significant values.

3 $$The\, biosynthetic\,\mathrm{ ZnO }\,NPs \,dry\, weight\, \left({g}/L\right)=15.6-0.021\, biosynthesis \,temperature-0.527 \,pH-0.0078 \,Agitation+0.136\, Reductant\, conc. - 10.91 \,Capping\, agents \,conc$$

The main effects describe the average variances in each variable between high and low values (Fig. 5A). When the main effect of a factor is positive, as in reductant conc. and capping agents, the reaction grows as the factor is increased from low to high. In contrast, the state lines for biosynthesis temperature, pH, and agitation varied from high to low, demonstrating the impact of a low value on the boosting reaction (Fig. 5A). The impact of different factors investigated in PBD was described using a Pareto Chart highlighting the order of significance of factors involved in bio-synthesized ZnO NPs (Fig. 5D). The key parameters regulating ZnO NPs' dry weight, as proved by the PBD, were reductant and capping agent levels, followed by pH and agitation (Table 4). The following are the main steps that mainly consist of the optimized biosynthesis procedure (Fig. 5C): Intracellular and extracellular fractions (v/v) were combined to prepare the reductant agent (cell-free extract), whose pH was then adjusted to 5 with NaOH before being titrated with 100 mM of ZnSO_4_. 7H_2_O (the precursor). This reaction was heated to 30 °C and stirred at 50 rpm for 15 min. Consequently, the presence of white color indicates the biosynthesis of ZnO NPs. To control aggregation, the excess mixed cell-free extract (25 mL) was then added as a capping agent to the generated ZnO NPs suspension (75 mL). This biosynthetic ZnO NPs pellet was collected by centrifuging it for 10 min at 10,000 rpm and then washing it with Milli-Q water several times. To yield powdery ZnO NPs, this cleaned pellet was then dried for a 24-h period at 60 °C. Using these optimized biosynthesis steps, the actual biosynthetic ZnO NPs yield (18.76 g/L) was increased by 4.3 times in comparison to the baseline (4.36 g/L). Additionally, the predicted biosynthetic ZnO NPs yield using these optimized biosynthesis steps was found to be 19.43 g/L (Fig. 5C). As can be seen, the verification revealed that our model had been validated with a high level of model accuracy of 96.54%.

### Scaling-up bioprocessing strategies for the cell mass of strain E56 and its biosynthetic ZnO NPs dry weights

**Figure 6:**
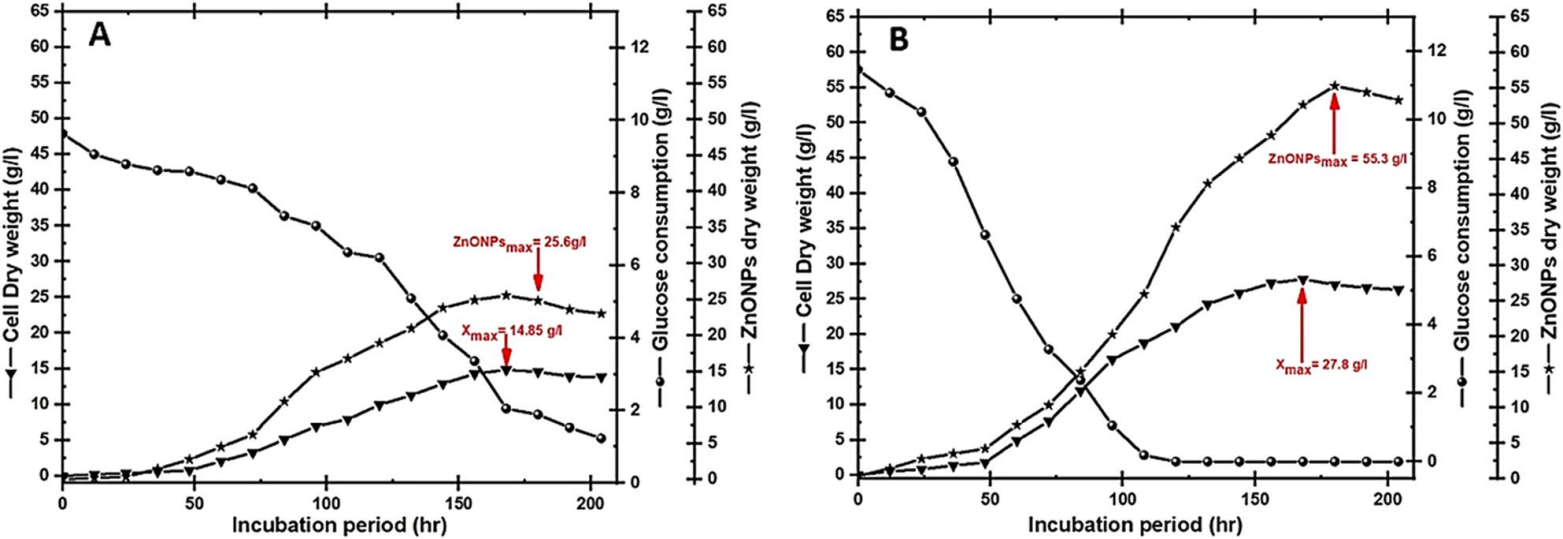
Represents the yields of the strain E56, biosynthetic ZnO NPs, and glucose consumption in two different fermentation programs: a 2-L flask system (**A**) and a 10-L stirred tank bioreactor (**B**) using batch-fermentation mode.

In fact, a number of microbial growth factors should be taken into consideration when developing the optimal microbial production line. Tools like fermentation modelling and chemometric analysis can be used to characterize chemical and physical parameters in near real-time using details from both online and offline measurement instruments^65^. To minimize the number of crucial tests that might be required when using large-scale cultures in a bioreactor, the best bioprocess methodology is first determined and developed on a small-scale (shake flasks). The scale-up strategy is a complex biochemical process that needs to be fine-tuned to provide unique microbial output at low production costs while also increasing efficiency^66^. This strategy is scaled from a small flask to a pilot scale to achieve the desired output and generate a significant quantity of these items. Many parameters, such as nutrient source availability, metabolite compartmentalization, agitation, aeration, fermenter broth capacity, and sterilization method, should be considered when switching scale size or fermentation modes^43^. Because of their effectiveness in optimizing a scaling-up strategy and achieving final product quality, these parameters should be successfully studied. The flask scale stage (mini-fermenter) is a great starting point whenever studying the variability of raw materials (inorganic, organic, or mixed), culturing procedures, temperature ranges, pH, inoculum size, and microbial sensitivities to high/low agitation conditions^67^. Bioreactors are an advanced version of the shake flask system with the option of a computer monitoring gas supply, pH settings, and feeding nutrients, and these data may be utilized to assist further evaluation in bigger bioreactor containers^44^. So, in our work, we employed batch fermentation mode with a 2-L flask as well as a 10-L bioreactor to gradually scale up biomass output in order to find the ideal growth production line (see Fig. 6). Cell growth kinetics were examined during batch cultivation of endophytic *Streptomyces* *albus* strain E56 in both a 2-L flask and a 10-L bench-top stirred tank bioreactor using the same medium composition with the ideal glucose level (40%).

Initially, the culture was propagated in a 2-L flask system (uncontrolled-pH mode) with fixed aeration (0.25 v/v), and a constant rate of agitation (100 rpm). According to Fig. 6A, the grown cells increased exponentially with a specific growth rate (µ = 0.05/h) throughout the lag phase (50 h). Consequently, the produced X_max_ was increased to 14.85 g/L, higher than Taguchi's result of 9.88 g/L. Therefore, compared to Placket’s result of 18.76 g/L, the biosynthetic ZnO NPs (P_max_ = 25.6 g/L) were increased by 1.4 times.

Second, to examine the impact of aeration and agitation parameters, scaling-up cultivation was accomplished in a 10-L controlled-bioreactor via batch fermentation mode. After nearly 120 h of the log phase, the glucose that serves as a function of cell growth in this culturing system was completely depleted. Additionally, in this phase, the DO level had gradually decreased to almost 40% saturation at 180 h. After the cells entered the stationary phase, the DO level was gradually raised until it reached about 89% saturation at the end of the cultural period. As a result of the combined effects of shear stress and glucose deficiency, the quantity of produced cells was significantly reduced. The controlled factors of aeration, agitation, and DO, with an uncontrolled pH, led to an increase in the X_max_ from the flake's 14.85 g/L to the bioreactor's 27.8 g/L, as shown in Fig. 6B. Furthermore, the maximum dry weight of biosynthetic ZnO NPs in the bioreactor was increased from the flask’s 25.6 g/L to 55.3 g/L.

**Figure 7:**
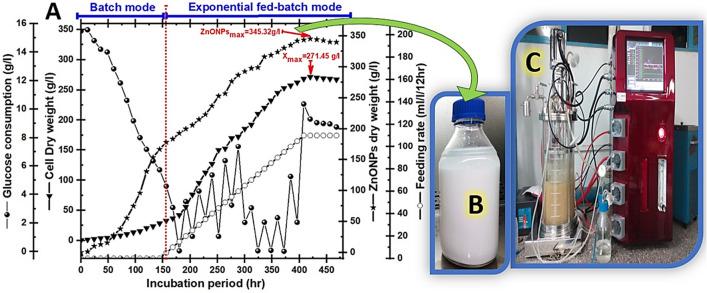
Represents (**A**) the yields of the strain E56, the biosynthesis of ZnO NPs (**B**), the feeding rate, and glucose consumption that were grown using fed-batch fermentation mode in a 10-L stirred Winpact bench-top fermentor (**C**).

Carbon substrate limitations, especially in *Streptomyces* may cause either breakdown or a reduction in growth production, depending on the structure and stability of the produced bioactive metabolites^44,66^. Some *Streptomyces* would be significantly destroyed if the quantity of produced metabolites was greatly increased, followed by a limitation on the carbon sources used in bioreactors. These microbial cells can continue to grow without entering a lysis phase if they can break down the produced bio-metabolites and use them for energy maintenance^68^. The rate of biomass production using batch fermentation mode may be significantly lower than that generated using fed-batch fermentation mode because there are not enough carbon sources available^65^. Therefore, as depicted in Fig. 7, the glucose level was controlled by employing an exponential pulse feeding regime with concentrated glucose solution to prolong microbial output and then biosynthetic ZnO NPs via a 10-L stirred tank bioreactor (Fig. 7C). After 156 h of cultivation, when the glucose concentration had reached about 3.08 g/L at the late log phase, feeding stages that required 5–110 mL/L/12 h were initiated. All microbial growth kinetics were determined to be as follows during fed-batch fermentation: the P_max_ yield was 345.32 g/L (Fig. 7B), the Y_X/S_ was 94.25 g/g, and the X_max_ was 271.45 g/L (Fig. 7A). Because all these fermentation conditions, including agitation, airflow, antifoam, and glucose concentration, were accurately controlled, the fed-batch model yields for calculated dry cell weight and biosynthetic ZnO NPs were higher than those achieved using other cultivation systems. To our knowledge, this report is the first to explain exactly how an exponential feeding system is employed to generate endophytic *Streptomyces* *albus* cells on a semi-industrial scale. Furthermore, this is the first study that scaled up the biosynthetic ZnO NPs yield using endophytic *Streptomyces* *albus* extract to a semi-industrial level.

### Characterization of the biosynthetic ZnO NPs

**Figure 8:**
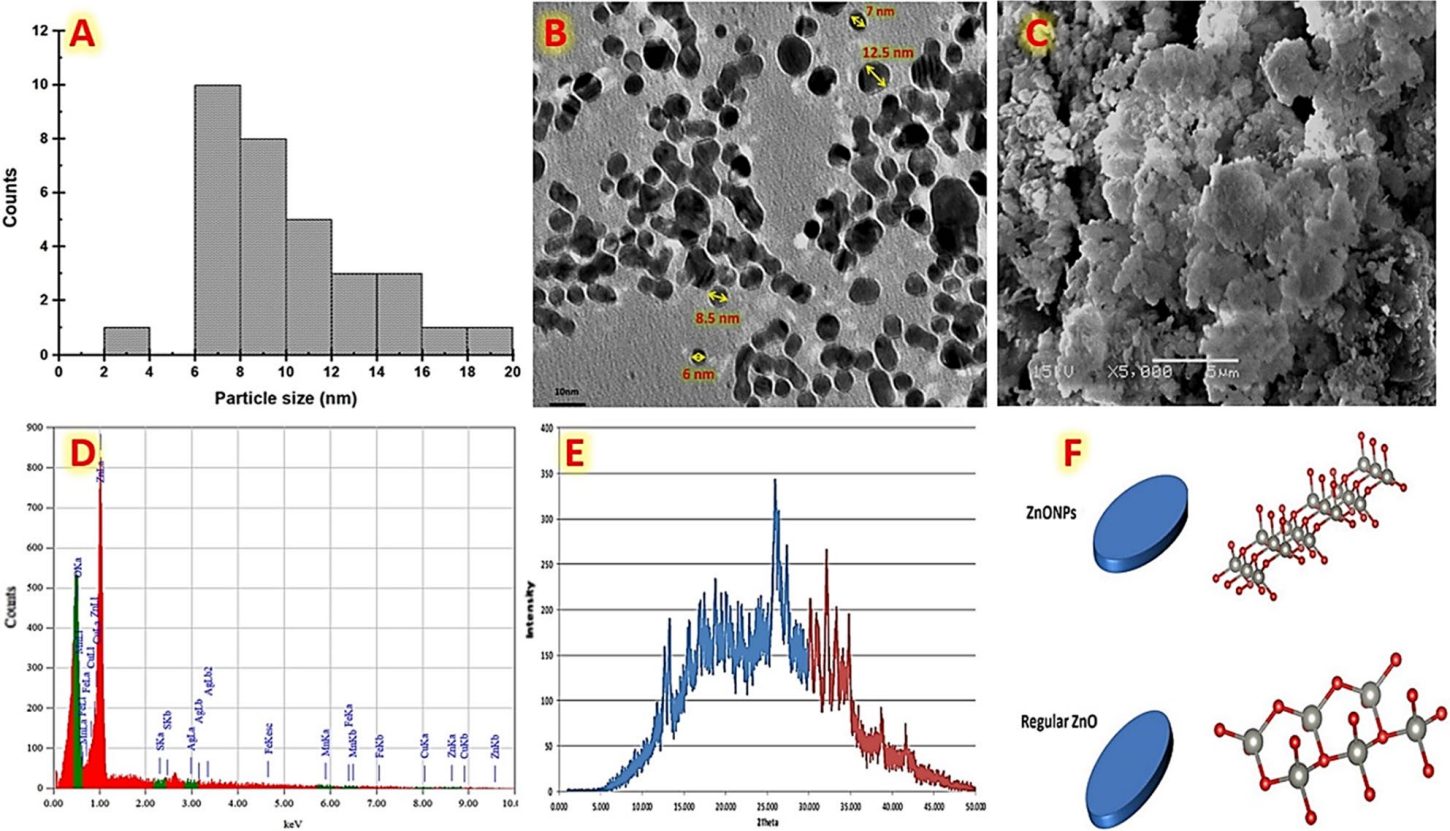
Represents the characterizations of the controllable biosynthetic ZnO NPs using mixed cell-free extract of endophytic *Streptomyces* *albus* strain E56 as a reductant/capping agent and ZnSO_4_.7H_2_O as a precursor; (**A**) the biosynthetic ZnO NPs particle size distribution; (**B**) TEM; (**C**) SEM; (**D**) EDX; (**E**) XRD; and (**F**) 3D model.

As described in this section, various tests were employed to characterize the biosynthetic ZnO NPs. The biosynthetic ZnO NPs size distribution histogram is shown in Fig. 8A, with an average particle size of 8 nm. Biosynthetic ZnO NPs are depicted in TEM and SEM micrographs in Fig. 8B,C, respectively. Our biosynthetic ZnO NPs were distinguished clearly by their uniform shape and semi-spherical morphology. It is indicated that a majority of the biosynthetic ZnO NPs have a flake-like structure. The crystallizing process has an impact on the particle size variation in the current observation. The large lateral flakes are the oldest crystal, while the tiny particles show early crystal formation. To evaluate the purity and chemical composition of the biosynthetic ZnO NPs, an EDX analysis was performed (Fig. 8D). The coexistence of Zn and O with foreign elements can be seen in the EDX spectrum, which shows that the chemical formula of our obtained NPs is Zn and O. Additionally, there are traces of some atoms that were found as impurities, which may have been connected to the used microbial extract. As seen in Fig. 8E, the biosynthetic ZnO NPs' XRD pattern is composed of two overlapped regions. The first region is distinguished by unpredicted crystalline structures, and undeclared peaks at approximately 2°: 17 and 27. This region may be refers to the organic components of the used microbial extract that attached on the surface of the biosynthetic ZnO NPs^69^. The second region is distinguished by characteristic peaks at 2: 39.244, 34.64, and 31.9 whish, which are indexed to the wurtzite hexagonal ZnO NPs crystalline phase with a space group of P63mc (186) and lattice parameters of a = 3.2498, b = 3.2498, and c = 5.2066 (ICDD card No. 00.036.1451). Additionally, the highest peak was seen at (2Ɵ: 0 ~ 32) indicating that our biosynthetic ZnO NPs formed in a direction along (100), while the majority of synthesized ZnO NPs developed in the (101)^70^. This configuration of our biosynthetic ZnO NPs may explain the formation of lateral flake-like particles. M. F. Elkady et al. previously generated ZnO NPs into diverse morphological shapes using a sol–gel method that developed in the (101) direction^71^. The XRD results were also employed to build a three-dimensional model of the biosynthetic ZnO NPs nanostructure using VESTA visualization package software. Figure 8F illustrates the hexagonal structure of the ZnO crystal, which contains four oxygen atoms arranged in a tetrahedron around the Zn atoms. Common wurtzite hexagonal ZnO NPs normally grow in the preferred growth direction (101) plane, but our current wurtzite hexagonal ZnO NPs were generated in the (100) direction. Accordingly, our work accurately describes the generation of biosynthetic ZnO NPs with a controllable growth direction along the (100) axis utilizing endophytic *Streptomyces* *albus*.

### Antimicrobial activities of the biosynthetic ZnO NPs

**Figure 9:**
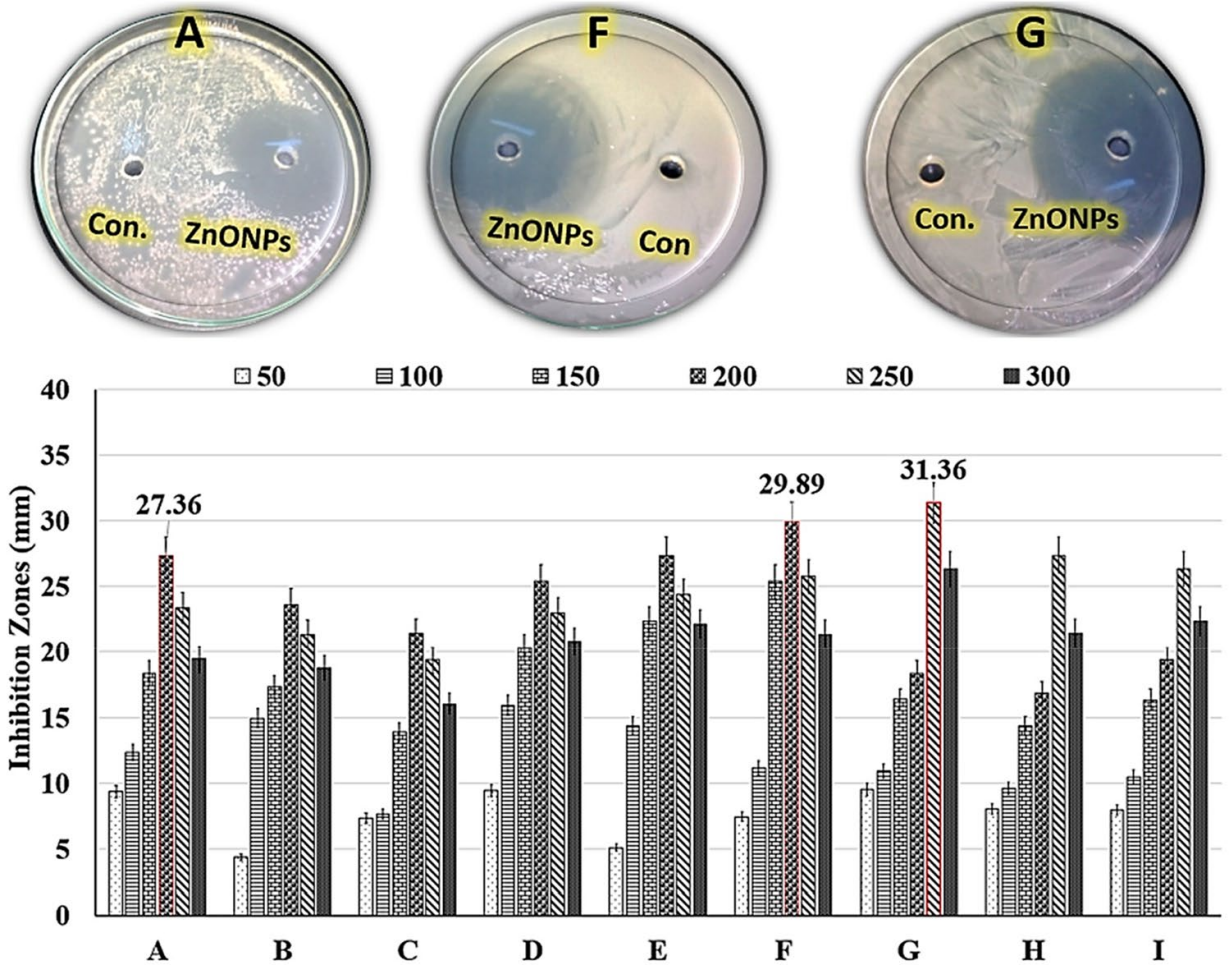
Antimicrobial activities of different doses of the controllable biosynthetic ZnO NPs (50, 100, 150, 200, 250, and 300 µg/mL) against some multi-drug resistant human pathogens, including: (**A**) *Escherichia* *coli*; (**B**) *Klebsiella* *pneumoniae*; (**C**) *Pseudomonas* *aeruginosa*; (**D**) *Staphylococcus* *aureus*; (**E**) *Salmonella* *typhimurium*, (**F**) *Streptococcus* *pneumoniae*, (**G**) *Candida* *albicans*, (**H**) *Candida* *krusei*; (**I**) *Candida* *tropicals*; and (con = control): endophytic *Streptomyces* *albus* E56 cell-free mixed cell-free extract.

Antibiotic resistance pathogens are becoming increasingly resistant to strong doses of several medications, causing difficulties such as unresponsiveness. The search for new chemicals in research is a difficult journey through millions of compounds. Another option is to employ existing approaches with minor modifications to give ample time to solve the problem^39^. Infections' ability to live in consortia is another paradigm to investigate, in which pathogens' biofilm ability creates a friendly environment for the destruction of normal tissue and disease progression. Making evolutionary modifications may be possible by genetically transforming the pathogen by changing the gene responsible. Rather, a basic extrovert chemical with a safe entitlement, such as ZnO NPs, can be very useful for biofilm destruction^20^. In our work, the effect of different dosages of the controllable biosynthetic ZnO NPs at various doses (50, 100, 150, 200, 250, and 300 µg/mL) against multi-drug resistant human pathogens was examined in vitro using the agar well diffusion method (Table 5). The zone of inhibition and concentration-dependent inhibition differentiation outcomes are shown in Fig. 9. At a dosage of 250 µg/mL, the maximum zone of inhibition was observed against *Candida* *albicans* (unicellular fungal cells), with a 31.36 ± 1.15 mm diameter. The remarkable inhibitory zones were detected in *Streptococcus* *pneumoniae* (gram + ve) and *Escherichia* *coli* (gram-ve) at 200 µg/mL concentrations, with 29.89 ± 1.37 and 27.36 ± 3.12 mm, respectively (Table 5). In addition, a 50 µg/mL concentration against *Klebsiella* *pneumoniae* produced a minimum zone of 4.32 ± 0.36 mm (Table 5). Furthermore, a concentration of 250 µg/mL is a rapidly expanding level. These results demonstrated that our biosynthetic ZnO NPs have concentration-dependent antimicrobial capabilities. Experts previously reported that increasing the concentration of LAB-ZnO NPs from 400 to 600 µg/mL increased their antifungal efficacy against *Candida* *albicans* (27 ± 0.50 mm at 600 µg/mL). In the case of *E.* *coli*, the lowest inhibition zone (7 ± 0.50 mm) was observed with 400 µg/mL acidophilus-ZnO NPs. Furthermore, *P.* *aeruginosa* was also the most sensitive to LAB-ZnO NPs (24 ± 0.57 mm)^72^.

**Table 5:** The antimicrobial activities of different dosages of the controllable biosynthetic ZnO NPs against some multi-drug resistant human pathogens.

Multidrug-resistant strains	The controllable biosynthetic ZnO NPs concentrations (µg/mL)						MIC (µg/mL)	MBC (µg/mL)	MFC (µg/mL)
	Inhibition zones (mm)								
	50	100	150	200	250	300			
*Escherichia* *coli*	9.76 ± 0.15	12.36 ± 1.36	18.36 ± 2.12	27.36 ± 3.12	23.36 ± 1.29	19.45 ± 1.45	75	80	
*Klebsiella* *pneumoniae*	4.32 ± 0.36	14.96 ± 2.64	17.36 ± 1.32	23.63 ± 1.19	21.36 ± 1.37	18.78 ± 1.23	70	80	
*Pseudomonas* *aeruginosa*	7.66 ± 0.96	7.69 ± 1.56	13.89 ± 1.89	21.45 ± 1.01	19.36 ± 1.48	16.23 ± 2.19	65	75	
*Staphylococcus* *aureus*	9.45 ± 0.31	15.96 ± 1.09	20.3 ± 0.87	25.36 ± 4.02	23.01 ± 1.67	20.75 ± 2.25	75	90	
*Salmonella* *typhimurium*	5.12 ± 0.24	14.36 ± 1.09	22.36 ± 1.25	26.36 ± 1.34	24.36 ± 2.38	22.1 ± 3.45	55	70	
*Streptococcus* *pneumoniae*	7.05 ± 1.56	11.15 ± 2.41	25.36 ± 3.56	29.89 ± 1.37	25.78 ± 1.09	21.36 ± 1.56	60	70	
*Candida* *albicans*	9.56 ± 2.36	10.96 ± 1.04	16.39 ± 2.15	18.36 ± 1.48	31.36 ± 1.15	26.36 ± 3.05	80		85
*Candida* *krusei*	8.05 ± 0.78	9.63 ± 0.07	14.36 ± 3.02	16.89 ± 3.56	27.36 ± 1.34	21.45 ± 2.12	85		90
*Candida* *tropicals*	7.96 ± 0.73	10.47 ± 1.56	16.36 ± 2.13	19.36 ± 2.71	26.36 ± 2.10	22.36 ± 2.75	95		90

The biosynthetic ZnO NPs' inhibitory concentration against biofilm-forming human pathogens was determined using spectrophotometric OD values, and the differentiation of concentration-dependent inhibition was also evaluated. When compared to the untreated control, the biofilms of all tested pathogens were not developed by using high doses of the biosynthestic ZnO NPs. As indicated in Table 5, the recorded MIC for all pathogens has ranged between 80 and 95 µg/L. Both bacterial and fungal pathogens' generated biofilms were absolutely absent (totally stopped) at concentrations greater than 90 µg/mL. For *Salmonella* *typhimurium*, the lower MIC was determined to be 55 µg/mL, whereas the MBC was reported to be 70 µg/mL. However, employing *Staphylococcus* *aureus*, which likewise had the highest MBC (90 µg/mL), as well as the highest MIC recorded at 75 µg/mL (Table 5).

The antimicrobial capabilities of NPs were influenced by their size, shape, concentration, surface area-to-mass ratio, and physico-chemical properties. For example, the size of NPs and their antimicrobial properties are inversely correlated; as the particle size decreased, the inhibitory effect increased^52^. Tiny NPs quickly interacted with the cell surface due to their increased surface reactivity, breaking the cell membrane and causing intracellular component leakage and cell death^33^. Furthermore, metal NPs can be directly attached to the thiol groups of cysteine amino acids rather than the sulfur of proteins, causing a significant change in the active sites and cell death^17^. The antimicrobial property of nanomaterials can be attributed to toxic metal ions created during metal termination and oxidative stress caused by the generation of reactive oxygen species (ROS) on the nanomaterials' surface. Nanomaterials with a positive surface charge can bind to pathogens with a negative surface charge, enhancing their antimicrobial capabilities. Furthermore, the particle size and type of material utilized to make NPs are two critical criteria in determining how effective they are against pathogens^73^. Previously, many nanostructures such as CuO, Mn_3_O_4_, AgO, ZnO, and TiO have all been extensively studied as potential antimicrobial systems^74–76^. Antimicrobial capabilities of ZnO nanostructures have been demonstrated against a variety of pathogenic species that can withstand both high temperatures and pressure^77,78^. Based on the above-mentioned mechanisms, our obtained results demonstrated that biosynthetic ZnO NPs at 70–90 µg/mL had anti-biofilm abilities and caused significant damage to internal and extracellular microbial components (Table 6).

**Table 6:** IC_50_, EC_100_ (μg/mL) and SI values of the biosynthetic ZnO NPs against normal (HFB-4) cells and tumor (A549, Caco-2, HepG-2, and MDA) cells after treatment for 24 h and 48 h.

Time	Value	HFB-4	A549	Caco-2	HepG-2	MDA
24 h	IC_50_	358.6 ± 14.31	67.26 ± 3.33	74.27 ± 4.18	59.03 ± 2.32	66.88 ± 5.07
	EC_100_	7.73 ± 0.71	1.39 ± 0.12	1.88 ± 0.16	1.71 ± 0.15	2.12 ± 0.22
	SI	–	5.33 ± 0.66	4.83 ± 0.58	6.07 ± 0.37	5.36 ± 0.64
48 h	IC_50_	259.6 ± 12.67	55.4 ± 4.48	51.03 ± 2.57	41.6 ± 3.67	58.05 ± 5.92
	EC_100_	4.25 ± 0.44	1.63 ± 0.16	0.79 ± 0.11	1.32 ± 0.15	1.84 ± 0.23
	SI	–	4.69 ± 0.61	5.09 ± 0.42	6.24 ± 0.34	4.47 ± 0.29

All values were expressed as mean ± SD.

### In vitro anticancer activity of the biosynthetic ZnO NPs

**Figure 10:**
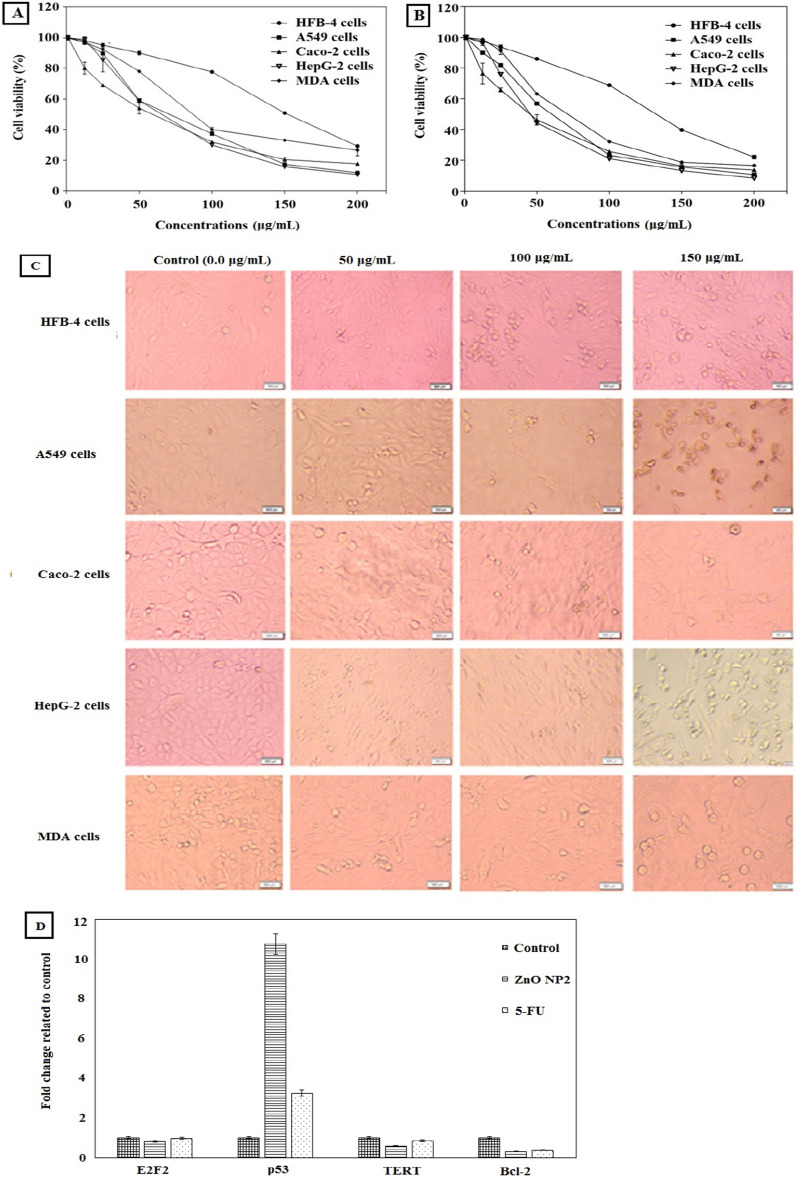
In vitro evaluation of anticancer activity of the prepared ZnO NPs. (**A,B**) Effect of the biosynthetic ZnO NPs at various concentrations (0–200 μg/mL) on the proliferation of normal (HFB-4) cells and tumor (A549, Caco-2, HepG-2, and MDA) cell lines after 24 h and after 48 h of treatment, respectively. (**C**) Effect of the biosynthetic ZnO NPs on morphological changes of normal (HFB-4) cells and different types of tumor cells including A549, Caco-2, HepG-2, and MDA cell lines under phase contrast microscope. Normal and tumor cells were treated with the biosynthetic ZnO NPs at different ratios of 50 μg/mL, 100 μg/mL and 200 μg/mL for 48 h as compared to reference control cells (0.0 μg/mL). (**D**) Effect of the biosynthetic ZnO NPs on relative changes in the expression levels of four key genes in HepG-2 cells after treatment in comparison with 5-FU for 48 h. All values are expressed as mean ± SD and represent the average values from three experiments.

In general, assaying the cytotoxicity of definite drug is usually performed using MTT method to determine cell metabolic activity. Hence, the tetrazolium ring of MTT is metabolized by mitochondrial dehydrogenases of viable cells, cleaving it to form purple MTT formazan crystals. Subsequently, the values of viable cells absorbance that formed after dissolving of formazan crystals are associated with the number of viable cells^79–81^. In the present study, we tested the effect of the biosynthetic ZnO NPs against HFB-4 cells as a normal cell line and different types of cancer cells, including A549, caco-2, HepG-2 and MDA cell lines. The cell viability of all tested cells was evaluated before and after treatment with the biosynthetic ZnO NPs, the highest values of EC_100_ and IC_50_ against normal cells refer to the highest safety. As shown in Table 6, values of EC_100_ and IC_50_ for the biosynthetic ZnO NPs on normal cells were be higher about 3 times than cancer cells which indicated its selectivity. Also, Table 6 indicates that the biosynthetic ZnO NPs had an anticancer activity on A549-7, Caco-2, HepG-2and MDA cells after treatment for 24 h at IC_50_ values determined to be 67.26 ± 3.33, 74.27 ± 4.18, 9.03 ± 2.32 and 66.88 ± 5.07, respectively, with SI values of 5.33 ± 0.66, 4.83 ± 0.58, 6.07 ± 0.37 and 5.36 ± 0.64, respectively. However, after treatment with the biosynthetic ZnO NPs for 48 h, values of IC_50_ were determined to be 55.4 ± 4.48, 51.03 ± 2.57, 41.6 ± 3.67 and 58.05 ± 5.92 against the same cell lines, respectively, with SI values of 4.69 ± 0.61, 5.09 ± 0.42, 6.24 ± 0.34 and 4.47 ± 0.29, respectively. In addition, Fig. 10A,B shows a significant increase in the anticancer effect of biosynthetic ZnO NPs, as well as high selectivity against all tested cancer cells while remaining safe for normal cells.

Figure 10C confirms the above results and reveals the proportional morphological investigation of four tested cancer cells before and after treatment with the biosynthetic ZnO NPs at concentrations of 50, 100 and 150 μg/mL for 48 h. Hence, all captured photomicrographs were observed in a live-cell mode via inverted phase contrast microscopy. All photomicrographs show that the treatment had a significant impact on all tested cancer cells. The images show that the biosynthetic ZnO NPs have a clear selectivity for cell damage and induce dose-dependent cell morphological changes. These cell changes involve shrinkage and blabbing as well as nuclear condensation (Fig. 10C). Consequently, based on these findings, it seems that the biosynthetic ZnO NPs enhance apoptotic pathways to provoke their anticancer effect. This apoptotic effect of the biosynthetic ZnO NPs might be exerted through complicated mechanisms that contribute to various cellular signaling modifications.

Effect of the biosynthetic ZnO NPs on E2F2, Bcl-2, TERT and p53 expression genes in HepG-2 cells was assessed by qPCR technique in comparison with 5-fluorouracile (5-FU) as a standard chemotherapy drug. Figure 10D shows that the gene expression of E2F2, Bcl-2 and TERT was suppressed in the biosynthetic ZnO NPs -treated HepG-2 cells more than 5-FU-treated HepG-2 cells. Gene expression level of Bcl-2 was obviously reduced in the treated cancer cells by more than 5 folds as compared to control cells. However, the level of p53 was evidently upregulated after treatment in HepG-2 cells by more than threefolds as compared to 5-FU-treated cells and by more than tenfolds as compared to untreated control cells. Thus, based on these in vitro results, it seems that the biosynthesized ZnO NPs provoke the apoptosis process to stimulate their antitumor activity against many different cancer cells. Our findings revealed the selectivity of the biosynthesized ZnO NPs toward different cancer cell lines, which has been demonstrated in several studies^82–85^. The biosynthesized ZnO NPs might disrupt the cellular membranes of the treated cells, generate vacuoles, modify the metabolism of the treated cancer cells, consequently stimulate the apoptosis pathway, and kill cancer cells. Besides the cellular and nuclear morphology modifications, ZnO NPs cause the release of the dissolved intracellular zinc ions, followed by generation of reactive oxygen species (ROS)^86^. Induction of ROS leads to damage of cellular membrane via protein denaturation and lipid peroxidation, consequently cell death by DNA damage and necrosis, resulting in cell killing by apoptosis process^87^. Apoptosis is supposed to be the major molecular mechanism of cell killing that caused as a response of ZnO NPs treatment ^88,89^. Hence, these novel biosynthesized ZnO NPs exerted a potent induction of apoptotic effect in different types of cancer cells.

## Conclusion

In this work, the Taguchi and Plackett–Burman designs were statistically assessed on a small scale to improve the biosynthetic ZnO NPs yield using endophytic *Streptomyces* *albus* strain E56 extract. Subsequently, in order to find the best industrial growing production line, we employed batch and fed batch fermentation modes to gradually scale up the biomass yields of *Streptomyces* *albus* strain E56 and ZnO NPs. As far as we are aware, this study provides a complete description of the semi-industrial production of endophytic *Streptomyces* *albus* cells employing an exponential feeding strategy. In this report, the generation of biosynthetic ZnO NPs with a controllable growth direction along the (100) axis has also been scaled up for the first time to a semi-industrial scale. This work suggests a new large-scale biosynthetic pathway for controllable biosynthetic ZnO NPs with effective biomedical potential, which could pave the way for industrial nanoparticle synthesis employing microbial platforms. Our findings provide strong support for the future use of the biosynthetic ZnO NPs in vivo as potential antimicrobial and anticancer agents with a low effect on normal tissues and cells.

## Materials and methods

### Materials

In this work the tested endophytic *Streptomyces* *albus* strain E56; (https://www.ncbi.nlm.nih.gov/nuccore/MF429779.1) as well as different multi-drug resistant human pathogens such as *Escherichia* *coli* (ATCC 10536), *Klebsiella* *pneumonia* (ATCC 10031), *Pseudomonas* *aeruginosa* (ATCC 27853), *Staphylococcus* *aureus* (ATCC 25923), *Salmonella* *typhimurium* (ATCC 13311), *Streptococcus* *pneumonia* (ATCC 33400), *Candida* *albicans* (ATCC 10231), *Candida* *krusei* (ATCC 6258), and *Candida* *tropical* (ATCC 13803) were kindly provided from Bioprocess Development Dep., Genetic Engineering and Biotechnology Research Institute (GEBRI), City of Scientific Research and Technological Applications (SRTA-City), New Borg Al-Arab City, 21934, Alexandria, Egypt.

### Biosynthesis of ZnO NPs using cell-free extracts of endophytic *Streptomyces albus* strain E56 and its antimicrobial survey

#### Pre-inoculum preparation

To cultivate the endophytic *Streptomyces* *albus* strain E56, three solutions should be prepared beforehand in order to prepare the NMM medium (minimal liquid medium). First, solution (A) which consisted of (NH_4_)_2_SO_4_; 2 g, Casamino acids; 5 g, MgSO_4_^.^7H_2_O; 0.6 g, and Polyethylene glycol 6000; 50 g was dissolved in 750 mL distilled water and sterilized by autoclave at 121 °C for 25 min. Secondly, the minor elements solution (solution B) containing 1 g ZnSO_4_^.^7H_2_O, 1 g FeSO_4_^.^7H_2_O, 1 g MnCl_2_^.^4H_2_O, and 1 g CaCl_2_ that dissolved in 1000 mL of distilled water and sterilized by filtration. The phosphate buffer (solution C), which was prepared by mixing 0.1 M NaH_2_PO_4_ and K_2_HPO_4_ to a pH of 6.8, was then autoclaved for 25 min at 121 °C. Lastly, the NMM medium was constructed by aseptically adding 750 mL of solution A, 1 mL of solution B, 124 mL of solution C, and 125 mL of sterilized glucose solution (20%). To prepare the pre-inoculum, this strain E56 was inoculated into the prepared NMM broth medium, then cultured for 3 days at 30 °C at 200 rpm^90^.

#### Biosynthesis of ZnO NPs

In a 250 mL conical flask, 9.40 g of ZnSO_4_.7H_2_O (Sigma-Aldrich) was stirred with 100 mL distilled water, then 10 g (wet weight) of the strain E56 were aseptically added. This cells suspension’s pH was adjusted to 8 with 1 M NaOH before being incubated for 96 h in a rotary shaker (150 rpm) at 28 °C. After incubation, this suspension color changed to a pale white, indicating the formation of ZnO NPs. Following that, the biosynthetic ZnO NPs were centrifuged at 13,000 rpm for 10 min, rinsed with deionized water, and then ethanol was used to remove the residual Zn^2+^. This pellet was collected and dried in a ceramic crucible in air atmosphere for 5 h at 500 °C. Finally, the biosynthetic ZnO NPs were collected and stored for further studies^33^. The maximal dry weights of produced cell mass and biosynthetic ZnO NPs were estimated by monitoring the growing time course throughout the cultivation period.

#### Pre-optimization steps

To prepare the cell free-extracts of strain E56; one mL of pre-inoculum was added to 50 mL of NMM broth medium, which was then agitated at 200 rpm for 96 h at 30 °C. To collect the pellet, this broth culture was centrifuged at 10,000 rpm for 10 min at 4 °C. This pellet was twice washed with sterile Milli Q water before being centrifuged at 4 °C for 25 min at 10,000 rpm. The obtained pellet was re-suspended in 5 mL of sterile Milli Q water, incubated for 24 h at 28 °C, then centrifuged for 10 min at 13,000 rpm at 4 °C to get the extracellular extract^91^. Subsequently, the intracellular extract was obtained by sonicating the collected cells twice for 1.5 min each time with Milli Q at 600 kHz and 50% maximum power^92^. The extracellular and intracellular fractions were used as a reductant and a capping agent in the biosynthesis of ZnO NPs. In addition, the mixed fraction (v/v) of external and intracellular fractions was examined as a reductant and a capping agent in the biosynthesis of ZnO NPs. To maximize the yield of biosynthetic ZnO NPs (g/L), a variety of precursors, including Zn (NO_3_)_2_.6H_2_O, ZnSO_4_.7H_2_O, and ZnCl_2_ at various concentrations were evaluated with different cell-free extracts. Then, the biosynthesis of ZnO NPs was confirmed using UV–visible spectroscopy in addition to the altered color. Then Fourier transform infrared spectroscopy (FTIR) was used to identify the functional groups in the efficient free-cell extract as well as the biosynthesized ZnO NPs.

#### Statistical optimization for strain E56 biomass production

In order to achieve a reliable outcome, the Taguchi technique was set up in stages, including the selection of significant factors, the building of an accurate matrix, the statistical analysis of the data, and validation using the optimum values^39,40^. The goal of this study was to get the largest achievable cell dry weight of strain E56 (g/L) by varying the concentrations of some medium ingredients such as (NH_4_)_2_SO_4_, Casamino acids, MgSO_4_^.^7H_2_O, Polyethylene glycol (PEG 6000), minor element solution (solution B), and glucose. For this optimization technique, the L_27_(3^6^) Taguchi orthogonal array design was adopted (6 factors, 3 levels, and 27 runs). The factor levels (inner array) are identified with numbers (1, 2, 3,…, and so on) and then evaluated against various noise factor combinations in the outer array, resulting in the construction of an orthogonal array (signal-to-noise ratio "S/N"). A decibel scale is used to express the S/N ratio [dB]. The F test and ANOVA are used to investigate the significance of all factors and their relationships at specific levels using the MINITAB 18 software after calculating the average of produced cell dry weights and the signal-to-noise (S/N) ratio (the larger the better group) for each process condition as designed. Finally, in a confirmation test, the obtained result using Taguchi's method were compared to the actual experimental value. The S/N ratio is measured in decibels [dB] and was computed using Eq. (4), where *n* is the number of observations and *Y* is the measured data (biomass yield). The predicted S/N ratio was calculated using Eq. (5), where S/N_m_ is the total mean S/N ratio and S/N_i_ is the mean S/N ratio at the ideal level; *n* is the number of parameters.

4 $$\frac{S}{N}ratio\, \left[\mathrm{dB}\right]=-10\, log\left[\frac{1}{n}\right]\sum_{i=1}^{n}\frac{1}{{Y}_{i}^{2}}$$

5 $$Predicted\, \left(\frac{S}{N}\right)ratio\, \left[\mathrm{dB}\right]=\frac{S}{{N}_{m}}+\sum_{i=1}^{n}\left[\frac{S}{{N}_{i}}-\frac{S}{{N}_{m}}\right]$$

#### Plackett–Burman design for optimization of the biosynthetic ZnO NPs yield

This design was used to evaluate the biosynthesis rate and final yield of ZnO NPs dry weight in several investigations. In these qualitative and quantitative screening tests, biosynthesis reaction conditions, including temperature (°C), pH, and agitation (RPM), as well as biosynthesis reaction ingredients such as reductant conc. (%), and capping agent conc. (v/v), were used to determine the best conditions for optimizing ZnO NPs dry weight. These factors were investigated at two levels: the highest (1) and the lowest (–1). All of the experiments were repeated twice, and the average cell dry weight was used to determine the response. For mathematical modelling, the PBD is based on a first-order polynomial model, as illustrated in Eq. (6). Where Y represents the dry weight of the biosynthetic ZnO NPs (Response), β_0_ indicates the model intercept, β_i_ symbolizes the linear coefficient, and X_i_ represents the level of independent variables. Furthermore, the efficiency of each variable was determined using Eq. (7), in which E_xi_ is the variable main effect, Mi+ and Mi– are the cell dry weights in trials where the independent variable (X_i_) was present at high and low levels, respectively, and N is the number of trials divided by 2. For statistical analysis and graphing, Minitab® 18.1 software was employed to generate a set of twelve-trails. The analysis of variance (ANOVA) was used to evaluate the influence of each independent variable on the response, with P < 0.05 considered significant. The multiple correlation coefficient (R^2^) and adjusted R^2^ were used to evaluate the equation's fitness.

6 $$Y={\beta }_{0}+\sum {\beta }_{i}{X}_{i}$$

7 $${E}_{Xi}=\frac{\left(\sum {M}_{i+}-\sum {M}_{i-}\right)}{N}$$

#### Scaling-up strategy for maximizing produced cell mass and biosynthetic ZnO NPs dry weights

To maximize the final bioprocessing production model, this scaling-up process was examined using a 2-L flask and then a 10-L bioreactor. The complete cultivation was studied to estimate the time required for complete glucose consumption as well as to get the maximum dry weights of biomass and the maximum biosynthetic ZnO NPs by estimating the growth kinetics. To prepare a pre-inoculum (vegetative culture), 200 mL of optimized broth medium was sterilized in a 500 mL Erlenmeyer flask, then inoculated with 2 mL of frozen spore suspension of strain E56 and cultured for 72 h at 30 °C at 200 rpm. This pre-inoculum was used to inoculate a flask or a bioreactor at a concentration of 5% (v/v). The 2-L flask and 10-L bioreactor had working volumes of 1 L and 8 L, respectively.

In these investigations, batch and fed-batch fermentation modes were employed in a 10-L bench-top fermentor (Winpact Bench-Top Fermentor, FS-01-B Series, version: V2.4, Cleaver Scientific Ltd., USA). The culture's pH and dissolved oxygen (DO) levels were measured in real time using in situ sterilizable electrodes. It was possible to overcome substrate limitations by feeding glucose into a fed-batch experiment. In fed-batch culture, glucose was supplied using peristaltic pumps (Watson Marlow, Wilmington, MA, USA) connected to the main bioreactor supervisory control system. A fed-batch culture approach was designed using an exponential pulse feeding system. Lastly, the DO was held above 40% saturation using airflow and/or agitation speed. Periodically, samples were obtained and centrifuged for 15 min at 10,000 rpm. The supernatant was then taken and stored at -20 °C for glucose determination. The glucose concentrations in the fermentation broth were determined using a glucose kit (Biovision's glucose assay kit). Furthermore, the pellets were washed twice with distilled water, centrifuged, and dried in a vacuum oven at 60 °C before being used to compute cell dry weight. Finally, the optimized method was employed to produce the biosynthetic ZnO NPs, which were subsequently dried in an oven at 80 °C to determine their yields (g/L). The first order equation indicates that the rate of change in cell mass is equal to the quantity of viable cell mass at any given point during the exponential growth phase. The behavior of microbial biomass production can be assessed and described kinetically using various criteria such as maximal cell dry weight (X_max_), maximal specific growth rate (µ_max_), maximal yield of biosynthetic ZnO NPs (P_max_), and yield coefficient (Y_x/s_)^93^. This growth yield coefficient, which is the quantity of produced cell dry weight per amount of glucose consumed, was calculated using Eq. (8). The dry weight of all biosynthetic ZnO NPs generated during the fermentation was also calculated using Eq. (9). Finally, in order to maintain a constant specific growth rate, Eq. (10) was employed to determine the feeding rate^94^.

8 $${Y}_{X/S}=\frac{\Delta X}{\Delta S}=\frac{X-{X}_{0}}{{S}_{0}-S}$$

9 $${P}_{max}=\frac{\Delta P}{\Delta t}=\frac{{P}_{max}-{P}_{0}}{{t}_{max}-{t}_{0}}$$

10 $$F=\frac{\mu {X}_{0}{V}_{0}{e}^{\mu t}}{{S}_{0}{Y}_{x/s}}$$

#### Morphological and physico-chemical analysis of the biosynthetic ZnO NPs

The crystalline structure of the produced ZnO NPs was determined by X‐ray diffraction (XRD, Shimadzu‐7000, Japan). While morphological structure of the produced ZnO NPs was investigated using scanning electron microscopy (SEM, JEOL JSM-6360LA, Japan). The functional group of the biosynthetic ZnO NPs was scanned-using Fourier Transform Infrared spectroscopy (Shimadzu FTIR-8400 S, Japan).

### Antimicrobial efficacy of the biosynthetic ZnO NPs

#### Bioassay survey

The antimicrobial sensitivity of biosynthetic ZnO NPs was evaluated in vitro against a variety of multi-drug resistant human pathogens, namely *E.* *coli*, *Staphylococcus* *aureus*, and *Candida* *albicans*, using an agar-well diffusion method. In a brief, sterile nutrient broth (0.5% peptone, 0.5% NaCl, and 0.3% yeast extract) was used to generate individual culture inocula of collected pathogens, which were then incubated at 37 °C for 24 h. The individual culture suspension was used for the bioassay survey after incubation. On Muller Hinton agar medium (0.2% beef extract, 0.15% starch, 1.75% casein, and 1.7% agar), 5-mm diameter wells were cut out with a sterile well cutter. After that, 0.1 mL of each culture suspension was dispersed on the agar plates, and 50 μL of biosynthetic ZnO NPs were loaded into the wells. In a separate well on the same plate, 20 μL of cell-free extract was used as a control. The inoculation culture plates were kept at 4 °C for 5 h before being cultivated for 48 h at 37 °C in an incubator. The inhibitory zones that emerged after incubation were assessed ^95^.

#### Antimicrobial sensitivity of biosynthetic ZnO NPs at various doses

Using an agar-well diffusion method, the antimicrobial sensitivity of the controllable biosynthetic ZnO NPs at different doses (50, 100, 150, 200, 250, and 300 µg/mL) was tested in vitro against a variety of multi-drug resistant human pathogens, namely: *Escherichia* *coli*, *Klebsiella* *pneumonia*, *Pseudomonas* *aeruginosa*, *Staphylococcus* *aureus*, *Salmonella* *typhimurium*, *Streptococcus* *pneumoniae*, *Candida* *albicans*, *Candida* *krusei* and *Candida* *tropicals*. After incubation, the inhibitory zones that appeared were evaluated. The minimum inhibitory concentration (MIC) of the biosynthetic ZnO NPs against the chosen individual pathogens was established using lower doses ranging from 50 to 100 µg/mL. Individual test tubes were loaded with the appropriate concentrations of ZnO NPs after being filled with 2 mL of sterile, nutritious broth medium. Following that, 0.1 mL of each of the different cultures was inoculated and incubated for 48 h at 37 °C, 200 rpm. After the culture tubes had been incubated, the turbidity was examined. The MIC was determined by the absence of turbidity in test tubes containing low doses of ZnO NPs. By spreading 100 µl of each broth culture on sterile nutrient agar plates, a minimum bactericidal concentration (MBC) and a minimum fungicidal concentration (MFC) were determined (MFC)^17,20^. The plates were then incubated for 48 h at 37 °C. Both MBC and MFC plates showed no microbial growth at low ZnO NPs doses. The data was analyzed in Excel 2016 after each experiment was conducted three times.

#### Cytotoxic effect of the biosynthetic ZnO NPs on normal and cancer cell lines

Normal human lung fibroblast Wi-38 cell line was obtained from American Type Culture Collection (ATCC, USA) and used to evaluate the anticancer effect of ZnO NPs. The cytotoxicity of the biosynthesized ZnO NPs on both normal and cancer cells was assayed by using the rapid colorimetric method of hydrogen acceptor of MTT [3-(4,5-dimethylthiazol-2-yl)- 2,5 diphenyltetrazolium bromide] assay was carried out^80,96,97^. The normal HFB-4 (normal human melanocytes) cells and cancer A549 (human lung) cells, Caco-2 (human colon) cells, HepG-2 (human hepatoma) cells and MDA (human breast) cells were seeded into four sterile 96-well tissue culture microplates at concentration of 1 × 10^4^ cells/well and incubated using a culture DMEM medium (Lonza, USA) supplemented with 10% fetal bovine serum (FBS) in CO_2_ incubator for 24 h. After cells attachment, various doses (from 50 to 250 μg/mL) of ZnO NPs were added to each cell line and incubated in 5% CO2 incubator. After incubation for 24 h and 48 h, the debris and dead cells were removed by washing 3 times with fresh medium, then 200 μL of 0.5 mg/mL MTT solution (Sigma-Aldrich) was added to cells, and all plates were incubated for 2–5 h at 37 °C. MTT solution was substituted with 200 μL of DMSO and the optical density of viable cells was read at 570 nm using a microplate spectrophotometer (BMG LabTech, Germany). The half maximal inhibitory concentration (IC_50_) values of ZnO NPs were calculated using the software of GraphPad Prism 6.0. Values of the selectivity index (SI), which is defined as the ratio of the IC_50_ on normal human cells (HFB-4) versus the IC_50_ value of cancer cells were determined^81,98^. Furthermore, the cytotoxic effect of ZnO NPs at doses of 50, 100 and 150 μg/mL on the morphology of each cancer cell line was analyzed using inverted phase contrast microscopy (Olympus, Germany). All samples were tested in triplicate and compared with untreated cells as a negative reference.

#### Effect of ZnO NPs on the gene expression level

To determine the effect of ZnO NPs on the expression levels of Transcription Factor 2 gene (E2F2), oncogene (Bcl-2), Telomerase reverse transcriptase gene (TERT) and tumour suppressor gene (p53) in HepG-2 cells, the quantitative detection method was performed using real time PCR. In brief, total RNAs of untreated and treated HepG-2 cells with IC_50_ doses of ZnO NPs were extracted according to manual of Gene JET RNA Purification Kit (Thermo Scientific, USA). After cDNAs synthesis were performed using cDNA Synthesis Kit (Thermo Scientific, USA), qPCR was performed by master mix of SYBR green kit and using the following primers (Forward/Reverse): 5′-GCATCCAGTGGAAGGGTGTG-3′/5′-ACGTTCCGGATGCTCTGCT-3′ for E2F2 gene, 5′-TCCGATCAGGAAGGCTAGAGTT-3′/5′-TCGGTCTCCTAAAAGCAGGC**-**3′ for Bcl-2, 5′-TGGGCACGTCCGCAAG-3′/5′-GAGCTCTGCTCGATGACGAC-3′ for TERT gene and 5′-TAACAGTTCCTGCATGGGCGGC-3′/5′-AGGACAGGCACAAACACGCACC-3′ for p53 gene. The 2^−ΔΔCT^ equation was used to evaluate the change in each gene expression for untreated and treated hepatoma HepG-2 cells with ZnO NPs.

## Data Availability

All data generated or analyzed during this study are included in this published article.
